# Myocardial Infarction Without Standard Modifiable Risk Factors From 2025-2040: Forecast Analysis of Multinational, Population-Based Study

**DOI:** 10.1016/j.jacasi.2026.01.027

**Published:** 2026-03-20

**Authors:** Grace Cao, Nicholas Weight, Silingga Metta Jauhari, Bryan Chong, Muhammad Rashid, Yiming Chen, Yip Han Chin, Haryo Raden Himan, Mingshi Cai, Si Min Kuo, Hui Wen Sim, Sara Tyebally, Xinyi Seow, Bryan Neo, Jayanth Jayabaskaran, Lynette Teo, Mark Muthiah, Zhan Yun Patrick Lim, Syed Saqib Imran, David Foo, Hee Hwa Ho, Jonathan Yap, Jiang Ming Fam, Zijuan Huang, Weien Chow, Siang Chew Chai, Liang Zhong, Lohendran Baskaran, Jack Wei Chieh Tan, Khung Keong Yeo, Andie H. Djohan, Christopher Koo, Jason Chen, Gavin Ng, Koo Hui Chan, Poay Huan Loh, Adrian F. Low, Chi Hang Lee, Ping Chai, James Yip, Tiong Cheng Yeo, Huay Cheem Tan, Derek J. Hausenloy, Gemma A. Figtree, A. Mark Richards, Mark Yan-Yee Chan, Mamas A. Mamas, Nicholas W.S. Chew

**Affiliations:** a Yong Loo Lin School of Medicine, National University Singapore, Singapore; b Keele Cardiovascular Research Group, Centre for Prognosis Research, Institute for Primary Care and Health Sciences, Keele University, United Kingdom; c Department of Biostatistics, Cardiovascular Research Institute, National University Heart Centre, National University Health System, Singapore; d Department of Cardiology, National University Heart Centre, National University Health System, Singapore; e NIHR Leicester Biomedical Research Centre, University of Leicester, United Kingdom; f Ministry of Health Holdings, Ministry of Health, Singapore; g Policy, Research and Surveillance Division, Health Promotion Board, Singapore; h Division of Cardiology, Department of Medicine, Ng Teng Fong General Hospital, Singapore; i Department of Diagnostic Imaging, National University Hospital, Singapore; j Division of Gastroenterology and Hepatology, Department of Medicine, National University Hospital, Singapore; k National University Centre for Organ Transplantation, National University Health System, Singapore; l Department of Cardiology, Khoo Teck Puat Hospital, Singapore; m Department of Cardiology, Tan Tock Seng Hospital, Singapore; n NHCS Cardiology, Sengkang General Hospital, Singapore; o Department of Cardiology, Changi General Hospital, Singapore; p National Heart Research Institute Singapore, National Heart Centre Singapore, Singapore; q Cardiovascular Sciences Academic Clinical Program and Cardiovascular Metabolic Disorder Program, Duke National University of Singapore Medical School, Singapore; r Department of Biomedical Engineering, National University of Singapore, Singapore; s Department of Cardiovascular Medicine, National Heart Centre Singapore, Singapore; t Cardiovascular and Metabolic Disorders Program, Duke-National University of Singapore Medical School, Singapore; u National Heart Research Institute Singapore, National Heart Centre, Singapore; v The Hatter Cardiovascular Institute, University College London, London, United Kingdom; w Northern Clinical School, Kolling Institute of Medical Research, University of Sydney, Sydney, New South Wales, Australia; x Department of Cardiology, Royal North Shore Hospital, Sydney, New South Wales, Australia; y Christchurch Heart Institute, University of Otago, Otago, New Zealand; z Cardiovascular Research Institute, National University Heart Centre Singapore, Singapore; aa Institute of Population Health, University of Manchester, Manchester, United Kingdom

**Keywords:** acute myocardial infarction, case fatality, prevalence, standard modifiable risk factors

## Abstract

**Background:**

Standard modifiable risk factors (SMuRFs) are associated with increased risk of acute myocardial infarction (AMI). Patients with AMI in the absence of standard modifiable risk factors (SMuRF-less) have demonstrated excess mortality.

**Objectives:**

The study aims to forecast trends in prevalence and case fatality of SMuRF-less AMI in Singapore and United Kingdom.

**Methods:**

Data from the Singapore Myocardial Infarction Registry and the UK Myocardial Ischemia National Audit Project were used to construct Poisson regression models to predict the prevalence and case fatality rate of SMuRF-less AMI from 2025 to 2040.

**Results:**

From 2025 to 2040, SMuRF-less AMI cases are expected to contribute to larger proportions of total AMI cases in Singapore (5.8% [95% CI: 5.2%-6.4%] to 8.5% [95% CI: 6.2%-10.8%]) and United Kingdom (13.8% [95% CI: 13.0%-14.6%] to 16.9% [95% CI: 15.1%-18.7%]). The proportion of SMuRF-less AMI mortality of the total AMI mortality is set to decrease in the United Kingdom (11.3% [95% CI: 10.5%-12.1%] to 9.5% [95% CI: 7.7%-11.4%]), but increase in Singapore (6.3% [95% CI: 5.7%-6.9%] to 13.3% [95% CI: 10.9%-15.6%]). Men continue to bear the majority of SMuRF-less AMI prevalence, with the most rapid rise in young adults in Singapore (8.1%; 95% CI: 7.5%-8.7%) and middle-aged adults in the United Kingdom (2.3%; 95% CI: 1.5%-3.1%). The increase in SMuRF-less AMI prevalence is set to be more rapid in the overweight/obesity population (3.9%; 95% CI: 3.3%-4.5%).

**Conclusions:**

By 2040, SMuRF-less AMI will expand to a larger proportion of the AMI census in Western and Eastern populations. SMuRF-less AMI vulnerability will shift toward a younger, male-dominant demographic, with overweight/obesity as a dominant risk factor.

Acute myocardial infarction (AMI) is identified as the largest contributor to the global burden of cardiovascular disease (CVD).^1^^,^^2^ The prevalence of AMI has been forecasted to rise nearly 3-fold between 2025 to 2050.^3^^,^^4^ This is paralleled by the rising prevalence of standard modifiable cardiovascular risk factors (SMuRFs), such as smoking, hypertension, type 2 diabetes mellitus, and hyperlipidemia.^3^^,^^5^, ^6^, ^7^ However, AMI can occur even in the absence of standard modifiable cardiovascular risk factors (SMuRF-less),^8^, ^9^, ^10^ with a paradoxical 50% higher risk of early cardiovascular-related mortality following AMI compared with those with SMuRFs.^7^^,^^10^ This has serious public health implications, because the global prevalence of SMuRF-less AMI is estimated to be 11.6% among all AMI cases,^10^ with a growing trend in its prevalence over the past decade.^8^ The future projection of SMuRF-less AMI prevalence and case fatality is therefore important to consider.

Because this seemingly healthy population is often overlooked in clinical trials or guidelines, this has led to a global initiative in harmonizing the SMuRF-less definition and a call to action for streamlined research in the prevention and treatment of SMuRF-less AMI.^11^ By modelling historical nation-level registry data, this study is the first to construct comparative forecast analysis of SMuRF-less AMI prevalence and case fatality rates in Singapore and the United Kingdom from 2025 to 2040, which serve as representative populations from Eastern and Western societies respectively. Secondary study objectives include projection estimates of SMuRF-less AMI across unique population groups, stratified by age, sex, AMI type, and the presence of overweight/obesity.

## Methods

### Overview and definition

The study used the historical estimates of AMI prevalence and case fatality rates based on all patients presenting with AMI in Singapore and the United Kingdom, captured by the Singapore Myocardial Infarction Registry (SMIR) and the Myocardial Ischemia National Audit Project (MINAP), respectively. The SMIR is a national registry of all patients aged ≥15 years diagnosed with AMI who presented at public and private hospitals in Singapore from January 2010 to December 2018.^12^ To be maximally unselected and all-encompassing, AMI cases were identified from hospital discharge records, reimbursement claims, the national death registry, and laboratory records. Cases with troponin levels elevated above the 99th percentile identified by laboratories based in Singapore were screened for AMI and included into the SMIR if the diagnostic criteria were fulfilled. All identified cases underwent central verification by trained personnel at the SMIR, following which information on patient demographics, clinical characteristics, and outcomes were collected by trained research personnel using standardized data elements from the SMIR notification forms. The International Classification of Diseases-9th Revision-Clinical Modification code 410 was used to identify AMI cases from the data sources before 2012, whereas the International Classification of Diseases-10th Revision Australian Modification codes I21 and I22 were used for AMI cases diagnosed from 2012 onwards. Further assessments were performed to ensure that there was no artificial breakpoint with the coding switch in 2012 (Supplemental Material 1).

To ensure high quality of the data collected, regular internal audits have been performed. Results from previous studies have consistently showed that the registry achieved high inter-rater reliability and logic check (>95%) for all data items. SMIR data were combined with mortality data from the Registry of Births and Deaths to obtain accurate in-hospital mortality outcomes.^12^ The study received a waiver for informed consent from patients because it used deidentified data, and it was approved by the SingHealth Centralised Institutional Review Board (Reference No. 2016/2480).

For the England and Wales comparison, the MINAP was used, which is a prospective national registry of patients admitted to hospitals in the United Kingdom with an AMI.^13^^,^^14^ The MINAP data set comprises 130 variables, including baseline demographics and clinical characteristics, comorbidities, management strategies, pharmacotherapy, in-hospital clinical outcomes, and discharge diagnosis. Data were submitted by hospital clinical staff, and approximately 90,000 pseudonymized records annually were uploaded to the National Institute for Cardiovascular Outcomes Research. For calculation of AMI prevalence over the study periods, duplicate patient records from the same day were dropped according to National Health Service unique identifier.^13^^,^^14^ In-hospital mortality was examined in MINAP, and “acute myocardial infarction,” “subsequent myocardial infarction,” or “complications following myocardial infarction” were recorded on the patients’ Medical Certificate of Cause of Death for each year of data collection in Office of National Statistics data.

### Definition of standard modifiable risk factors

Individuals were diagnosed with AMI, which included both ST-segment elevation myocardial infarction (STEMI) or non–ST-segment elevation myocardial infarction (NSTEMI), by the attending cardiologists based on clinical evidence.^15^ All type 2 myocardial infarction cases were excluded from the analysis. The consensus-driven proposal of the harmonized definition of SMuRF-less status was used in this study.^11^ SMuRF-less individuals were defined as patients without any of the following risk factors: active/previous smoking, hypertension, type 2 diabetes mellitus, and hyperlipidemia (Supplemental Material 2). Notably, overweight/obesity was not included as 1 of the 4 SMuRFs within the harmonized SMuRF-less definition, because early evidence has suggested that adiposity may not be the main driver of advanced atherosclerosis in the SMuRF-less cohort.^10^^,^^11^ Other studies have not included overweight/obesity within the SMuRF definition, citing that body mass index (BMI) as a surrogate of overweight/obesity is a poor measure of high-risk body fat distribution.^7^^,^^10^^,^^11^^,^^16^, ^17^, ^18^, ^19^, ^20^, ^21^ Nevertheless, the present study has performed sensitivity analysis of the prevalence and case fatality rates of SMuRF-less AMI in individuals with overweight/obesity. Overweight/obesity was defined as a BMI ≥23 and ≥25 kg/m^2^ based on the World Health Organization threshold recommended for Asian and Western populations, respectively.^17^^,^^22^

### Statistical analysis

All statistical analyses were performed using STATA version 17.0 and R Studio 4.4.1. To predict the burden of AMI in the SMuRF-less population from 2025 to 2040, SMIR and MINAP data from 2010 to 2018 were used to calculate crude AMI cases and mortality. The study only included the first AMI event per individual in the analysis to ensure independence of observations. The prediction models for crude AMI cases and mortality in SMuRF-less, SMuRF-less overweight/obesity, and total AMI populations were generated using Poisson regression, with AMI onset year as the independent variable and annual number of AMI cases as the dependent variable. In addition, the exclusion of 2019-2021 data was to avoid pandemic-related distortions of the long-term projections. Sensitivity analysis was performed to include COVID-19–affected years, which demonstrated that long-term projections were not biased with consistent forecast trajectories across all models (Supplemental Material 3). Moreover, imputation models were applied to missing BMI data from the MINAP data set, with distribution checks performed to ensure no significant variability in the generated BMI across imputations. The study analysis imputed BMI as a monotone variable alone and created 10 imputed data sets in the initial imputation model. Although we used an imputed model that would typically undertake multiple imputation by chained equations (MICE), the missing-value pattern was monotone. As such, additional iterations were not performed, and monotone imputation was used (Supplemental Figure 1). We have provided a comprehensive missing BMI data analysis summarizing the extent and patterns of missingness across key variables used in the time-series construction and identifying those included in the imputation model (Supplemental Figure 1). The missing data mechanism was assumed to be missing at random, and our findings suggest that missingness was primarily associated with calendar time, an observed variable that was accounted for in the imputation model, rather than unobserved subgroup-specific mechanisms. This pattern supports the plausibility of the missing at random assumption underlying the multiple imputation procedure. Diagnostic assessments of the multiple imputation procedure are also provided in Supplemental Material 4, where forecasts derived independently from all 10 imputed data sets, as well as complete-case data analyses, demonstrate highly consistent trajectories with substantial overlap of the 95% CIs. The agreement across imputations and the concordance in forecast direction between imputed and complete-case analyses indicate that the primary conclusions are robust to the handling of missing BMI data and are not driven by the imputation procedure.

Poisson regression is a well-established method for modelling count data, particularly in epidemiological studies involving large national registries. The Poisson regression model was fitted to crude prevalence rates and crude mortality rates rather than the total event counts, ensuring that population growth and denominator effects were appropriately accounted for, mitigating population-driven variance. Goodness-of-fit (GoF) assessments were performed using Pearson and Deviance statistics, which showed no significant lack of fit (*P >* 0.005) for Singapore and UK data sets, indicating that the Poisson assumption is reasonable for modelling rate-based data over time. Assessment of overdispersion was performed using the dispersion parameter (ϕ), calculated as the ratio of the Pearson chi-square or deviance statistic to the corresponding residual df (ϕ = χ^2^/df). Dispersion values close to 1 indicate an appropriate Poisson fit, whereas values substantially >1 suggest overdispersion. Across all Poisson models, dispersion parameters were consistently well below 1 (range 0.03-0.05), with nonsignificant goodness-of-fit *P* values (*P >* 0.99), indicating no evidence of overdispersion and only mild under dispersion. This pattern is expected when modelling aggregated annual rate data rather than individual-level event counts, and this results in conservative SEs, thus supporting the robustness of the reported estimates. No population offset term was included in the Poisson regression models, because the outcome variables were already expressed as standardized rates per 100,000 population, thereby accounting for population denominators during rate construction.

To further evaluate predictive performance, we performed temporal validation by fitting the models using data from 2010-2015 and forecasting for 2016-2018. All analyses were conducted separately for the Singapore and UK data sets, with no pooled modelling across countries. Heterogeneity between countries was addressed by stratification at the analysis stage, with each country treated as a distinct analytical unit. This approach avoids imposing common model assumptions across populations with differing demographic structures and baseline disease risks. Extrapolation accuracy was then quantified using Mean Error (ME) and Root Mean Square Error (RMSE). To validate the performance of the Poisson regression model, we compared this to other established forecast models such as Negative Binomial and Autoregressive Integrated Moving Average. The Poisson and Negative Binomial models produced nearly identical RMSE and ME values, suggesting minimal overdispersion, confirming that the Poisson model was sufficient. Both models substantially outperformed the Autoregressive Integrated Moving Average model, which exhibited higher forecast errors and poorer extrapolation accuracy. As such, the Poisson regression model was chosen as the primary modelling framework for extrapolation, because it provides a parsimonious, stable, and empirically robust approach for forecasting AMI prevalence and mortality trends from 2025 to 2040 (Supplemental Materials 5 and 6). This modelling approach was applied to the entire cohort of patients with AMI, with further stratified analyses conducted across several subgroups: men, women, young adults aged 15-39 years, middle-aged adults aged 40-64 years, older adults aged ≥65 years, STEMI, NSTEMI, and the presence of overweight/obesity. Although several approaches were considered in computing the 95% CIs of the predicted prevalence and case fatality rates (such as bootstrap CIs, scenario-based Monte Carlo simulations, and Bayesian posterior prediction intervals), we chose the delta method for the estimation of forecast intervals (Supplemental Material 7).

The prevalence of SMuRF-less AMI was calculated using the crude SMuRF-less AMI cases over the crude total AMI cases. The case fatality rate of SMuRF-less AMI was derived from the crude SMuRF-less AMI mortality over the crude SMuRF-less AMI cases. The proportion of SMuRF-less AMI mortality was calculated with the crude SMuRF-less AMI mortality over the crude total AMI mortality. All values were represented as percentages. Compound annual growth rate (CAGR) was used to examine the annual rate of change of AMI prevalence and case fatality. CAGR was calculated using the formula:

$$CAGR=\left({\left(\frac{EV}{BV}\right)}^{\frac{1}{n}}-1\right)\times 100$$

Where EV represents ending value, BV represents beginning value, and n represents number of years.

## Results

### Overview

**Figure 1:**
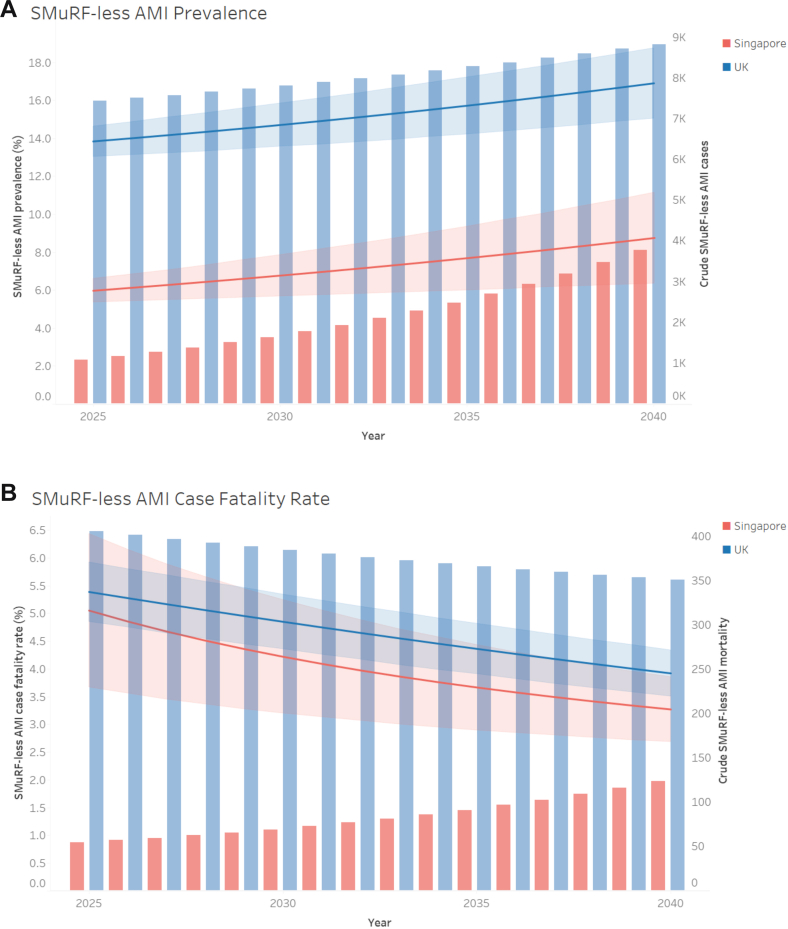
SMuRF-Less AMI Prevalence and Case Fatality Rate (A) Projected absence of standard modifiable risk factors (SMuRF-less) acute myocardial infarction (AMI) prevalence and (B) projected SMuRF-less AMI case fatality rate in Singapore and United Kingdom from 2025 to 2040. Bar charts depict crude SMuRF-less AMI cases (or mortality), and line graphs depict SMuRF-less AMI prevalence (or case fatality rate).

**Figure 2:**
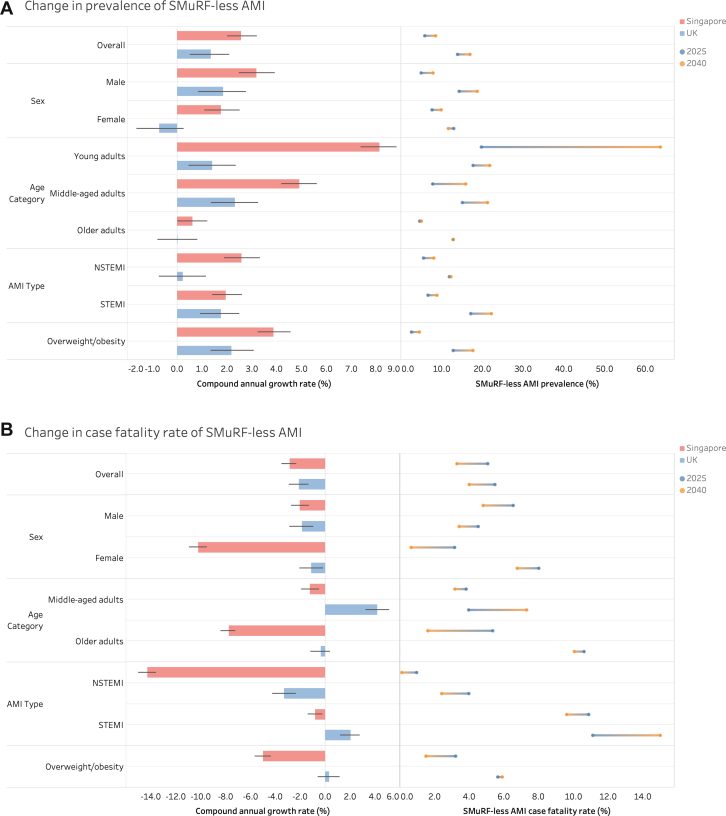
Change in Prevalence and Case Fatality Rate of SMuRF-Less AMI (A) Compound annual growth rate and SMuRF-less AMI prevalence and (B) compound annual growth rate and SMuRF-less AMI case fatality rate from 2025 to 2040. The young adult subgroup was omitted from B. Case fatality trend in the age 15-39 years category was not forecasted because of the low number of mortality cases. Abbreviations as in Figure 1.

Of the overall AMI population, the proportion of SMuRF-less AMI cases is expected to rise from 5.8% (1,073 of 18,523; 95% CI: 5.2%-6.4%) in 2025 to 8.5% (3,771 of 44,515, 95% CI: 6.2%-10.8%) by 2040 in Singapore, and 13.8% (7,427 of 52,659; 95% CI: 13.0%-14.6%) to 16.9% (8,820 of 52,174; 95% CI: 15.1%-18.7%) in the United Kingdom (Figure 1, Supplemental Table 1). The prevalence of AMI in the SMuRF-less population is anticipated to rise with a CAGR of 2.6% (95% CI: 2.0%-3.2%) in Singapore and 1.3% (95% CI: 0.5%-2.1%) in the United Kingdom (Central Illustration**,** Figures 1 and 2**,** Supplemental Table 2). The projected total AMI prevalence in Singapore and United Kingdom is summarized in Supplemental Figure 2.

**Central Illustration:**
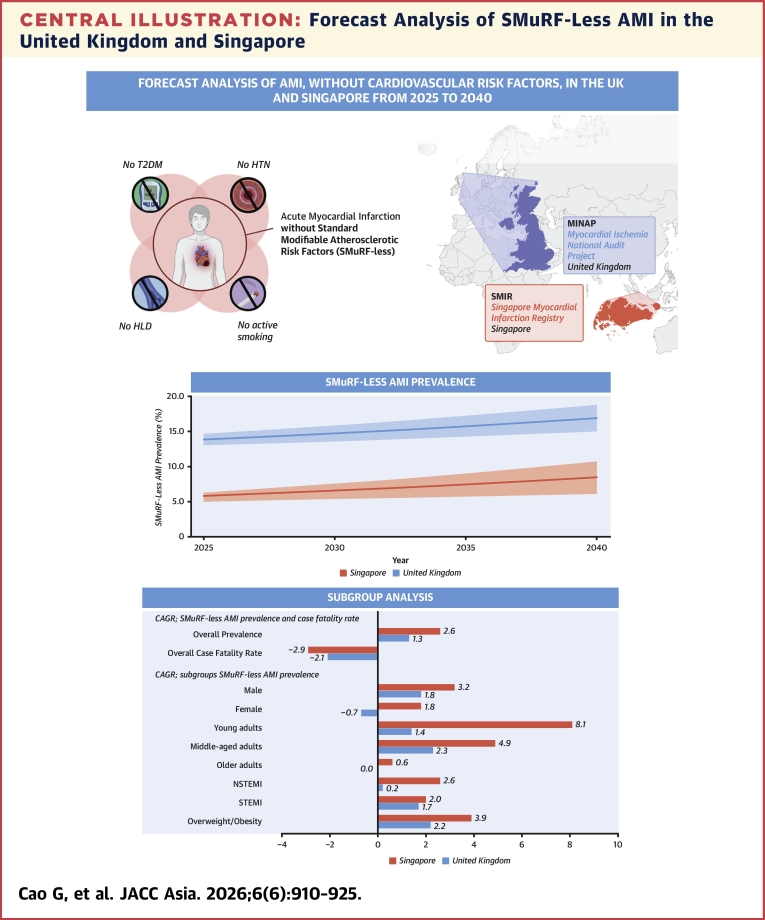
**Forecast Analysis of SMuRF-Less AMI in the United Kingdom and Singapore** Forecast analysis of acute myocardial infarction (AMI) without standard modifiable cardiovascular risk factors in the United Kingdom and Singapore from 2025 to 2040. Line graphs depict absence of standard modifiable risk factors (SMuRF-less) AMI prevalence in the United Kingdom and Singapore, and bar graphs depict change in SMuRF-less AMI prevalence (and case fatality rate) represented by compound annual growth rate, stratified by subgroups. CAGR = compound annual growth rate; HLD = hyperlipidemia; HTN = hypertension; NSTEMI = non–ST-segment elevation myocardial infarction; STEMI = ST-segment elevation myocardial infarction; T2DM = type 2 diabetes mellitus.

The proportion of SMuRF-less AMI mortality of the overall AMI mortality will likely see an increase from 6.3% (54 of 855; 95% CI 5.7%-6.9%) in 2025 to 13.3% (123 of 930; 95% CI: 10.9%-15.6%) by 2040 in Singapore, but a decrease from 11.3% (406 of 3,581; 95% CI: 10.5%-12.1%) to 9.5% (351 of 3,679; 95% CI: 7.7%-11.4%) in the United Kingdom (Supplemental Figure 3**,** Supplemental Table 1). SMuRF-less AMI case fatality rate in Singapore is predicted to decrease from 5.1% (54 of 1,073; 95% CI: 3.7%-6.4%) to 3.3% (123 of 3,771; 95% CI: 2.7%-3.9%; CAGR −2.9%; 95% CI: −3.5% to −2.3%), while SMuRF-less AMI case fatality rate in the United Kingdom will likely decrease from 5.5% (406 of 7,427; 95% CI: 4.8%-6.2%) to 4.0% (351 of 8,820; 95% CI: 3.6%-4.4%; CAGR −2.1%; 95% CI: −2.9% to −1.3%) (Figures 1 and 2, Supplemental Table 3).

### Sex differences in SMuRF-less AMI

**Figure 3:**
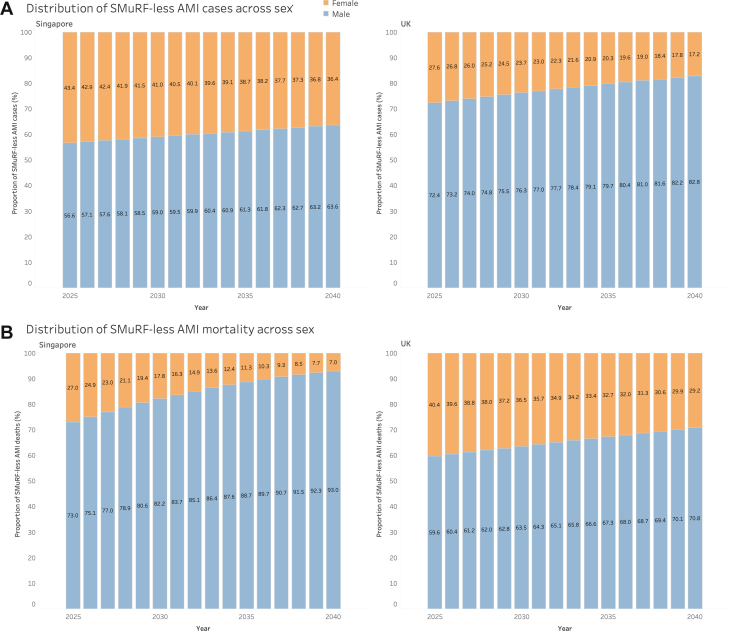
Distribution of SMuRF-Less AMI Cases and Mortality Across Sex (A) Proportion of men and women among all SMuRF-less AMI cases and (B) proportion of men and women among all SMuRF-less AMI mortality in Singapore and UK from 2025 to 2040. Abbreviations as in Figure 1.

**Figure 4:**
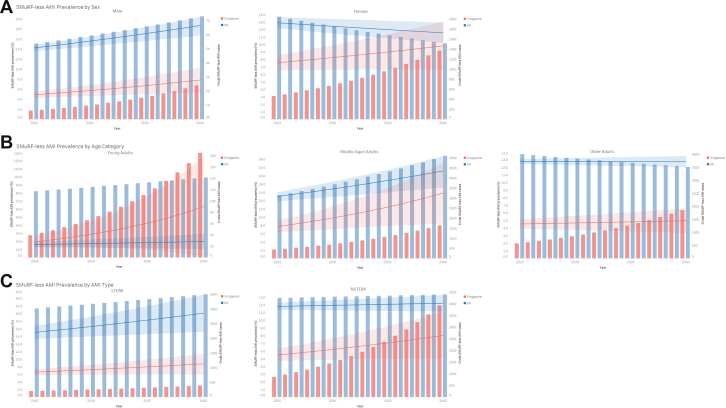
Projected SMuRF-Less AMI Prevalence, Stratified by Subgroups Bar charts depict crude SMuRF-less AMI cases, and line graphs depict SMuRF-less AMI prevalence in Singapore and the United Kingdom from 2025 to 2040, stratified by (A) sex, (B) age category, and (C) AMI type. NSTEMI = non–ST-segment elevation myocardial infarction; STEMI = ST-segment elevation myocardial infarction.

Men will continue to bear the majority of SMuRF-less AMI prevalence from 2025 to 2040 in both countries. Notably, there will be a wider sex-specific disparity observed in the United Kingdom compared with Singapore by 2040 (Figure 3). In the male population with AMI, SMuRF-less AMI prevalence is anticipated to increase to a larger extent in Singapore from 4.9% (607 of 12,387; 95% CI: 4.3%-5.5%) to 7.8% (2,400 of 30,591; 95% CI: 5.4%-10.3%; CAGR 3.2%, 95% CI: 2.6%-3.8%), compared with the United Kingdom (14.2% [5,378 of 37,791; 95% CI: 13.6%-14.8%) to 18.7% (7,304 of 39,078; 95% CI: 16.7%-20.4%); CAGR 1.8%; 95% CI: 1.0%-2.6%). On the other hand, the prevalence of SMuRF-less AMI in the female population with AMI is set to increase from 7.6% (465 of 6,136; 95% CI: 6.6%-8.6%) to 9.8% (1,371 of 13,924; 95% CI: 6.6%-13.1%; CAGR 1.8%; 95% CI: 1.2%-2.4%) in Singapore. This is in contrast to the forecasted decline in SMuRF-less AMI prevalence in the UK female population, from 12.9% (2,049 of 15,868, 95% CI: 12.2%-13.6%) to 11.6% (1,515 of 13,096; 95% CI: 10.2%-12.9%; CAGR −0.7%; 95% CI: −1.5% to 0.1%) (Figures 2 and 4, Supplemental Table 2).

Of the overall SMuRF-less AMI-related mortality, the proportion of the male population succumbing to SMuRF-less AMI mortality will continue to rise disproportionately (Figure 3). SMuRF-less AMI case fatality rate is set to decrease in men from 6.5% (40 of 607; 95% CI: 1.8%-5.1%) to 4.8% (115 of 2,400, 95% CI: 0.1%-8.7%) in Singapore (CAGR −2.0%; 95% CI: −2.6% to −1.4%) and to a smaller extent in the United Kingdom from 4.5% (242 of 5,378; 95% CI: 3.7%-5.3%) to 3.4% (248 of 7,304; 95% CI: 0.1%-14.7%; CAGR −1.9%; 95% CI: −2.7% to −1.1%). This decreasing trend is also observed in the female population, with a more rapid decline predicted in Singapore from 3.1% (15 of 465; 95% CI: 0.8%-3.7%) to 0.6% (9 of 1,371; 95% CI: 0.1%-1.2%; CAGR −10.2%; 95% CI: −10.8% to −9.6%), compared to that in the United Kingdom (8.0% [164 of 2,049; 95% CI: 7.2%-8.8%] to 6.8% [102 of 1,515; 95% CI: 4.3%-11.5%); CAGR −1.1%; 95% CI: −1.9% to −0.3%) (Figure 2, Supplemental Figure 4, Supplemental Table 3).

### Age profiles in SMuRF-less AMI

In Singapore, the largest burden of SMuRF-less AMI will be borne by the older adults (aged ≥65 years), followed by middle-aged (aged 40-64 years), and young adults (aged 15-39 years). In the United Kingdom, the age category of patients bearing the majority of SMuRF-less AMI prevalence will transition from older adults to the middle-aged adults by year 2032 (Supplemental Figure 5). An increase in SMuRF-less AMI prevalence will be observed across all age categories in both countries from 2025 to 2040, with large increases seen in young adults (CAGR 8.1%; 95% CI: 7.5%-8.7%; increase in 146 cases) and the middle-aged adults (CAGR 4.9%; 95% CI: 4.3%-5.5%; increase in 1,206 cases) in Singapore. In the United Kingdom, a larger increase in SMuRF-less AMI prevalence will be seen in middle-aged adults (CAGR 2.3%; 95% CI: 1.5%-3.1%; increase in 248 cases) followed by young adults (CAGR 1.4%; 95% CI: 0.6%-2.2%; increase in 25 cases). The older adults are projected to have the smallest increase in SMuRF-less AMI prevalence for both countries (Figures 2 and 4, Supplemental Table 2).

SMuRF-less AMI case fatality rates are expected to decrease in the middle-aged adult population over the years, from 3.8% (17 of 449; 95% CI: 3.4%-7.0%) to 3.2% (52 of 1,655; 95% CI: 10.4%-26.0%; CAGR −1.2%; 95% CI: −1.8% to −0.6%) in Singapore, but rise from 3.9% (124 of 3,139; 95% CI: 3.2%-4.7%) to 7.3% (372 of 5,102; 95% CI: 2.9%-26.9%; CAGR 4.2%; 95% CI: 3.4%-5.0%) in the United Kingdom. Case fatality rates are set to decline in the older adults, from 5.3% (31 of 581; 95% CI: 3.5%-21.7%) to 1.6% (30 of 1,861; 95% CI: 1.4%-20.7%; CAGR −7.8%; 95% CI: −8.4% to −7.2%) in Singapore but remain relatively constant in the United Kingdom (10.6% [425 of 4,003; 95% CI: 10.0%-11.2%] to 10.0% (353 of 3,515; 95% CI: 9.1%-16.7%); CAGR −0.4%; 95% CI: −1.2% to 0.4%) (Figure 2, Supplemental Figure 6, Supplemental Table 3).

### SMuRF-less AMI types

**Figure 5:**
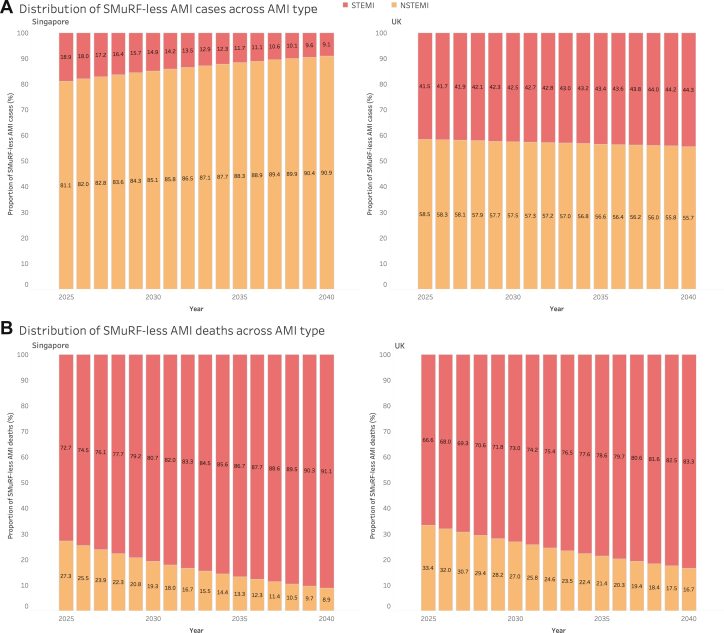
Distribution of SMuRF-Less AMI Cases and Mortality Across AMI Type (A) Proportion of projected SMuRF-less AMI cases and (B) proportion of projected SMuRF-less AMI mortality in Singapore and the United Kingdom from 2025 to 2040, stratified by AMI type. Abbreviations as in Figures 1 and 4.

From 2025 to 2040, NSTEMI presentations will be the dominant SMuRF-less AMI type, with a larger disparity in AMI types observed in Singapore compared with the United Kingdom (Figure 5). The prevalence of SMuRF-less NSTEMI will rise from 5.5% (870 of 15,839; 95% CI: 4.7%-6.3%%) to 8.0% (3,929 of 48,838; 95% CI: 5.1%-11.1%; CAGR 2.6%, 95% CI: 2.0%-3.2%) in Singapore but remain relatively constant in the United Kingdom (11.8% [4,255 of 35,960; 95% CI: 11.4%-12.3%) to 12.2% (4,403 of 35,962; 95% CI: 11.2%-13.3%); CAGR 0.2%, 95% CI: −0.6% to 1.0%). SMuRF-less STEMI is forecasted to increase from 6.6% (202 of 3,071; 95% CI: 5.8%-7.3%) to 8.8% (392 of 4,457; 95% CI: 6.0%-11.4%) in Singapore (CAGR 2.0%; 95% CI: 1.4%-2.6%), and from 17.1% (3,024 of 17,703; 95% CI: 15.4%-18.7%) to 22.1% (3,508 of 15,836; 95% CI: 17.2%-27.0%) in the United Kingdom (CAGR 1.7%; 95% CI: 0.9%-2.5%) (Figures 2 and 4, Supplemental Table 2).

Over the coming years, there will be an increasing proportion of mortality related to SMuRF-less STEMI from 72.7% (22 of 30) of all SMuRF-less AMI mortality to 91.1% (38 of 42) in Singapore, and 66.6% (336 of 504) of all SMuRF-less AMI mortality to 83.3% (526 of 631) in the United Kingdom (Figure 5). SMuRF-less STEMI and NSTEMI case fatality rates can be found in Figure 2, Supplemental Figure 7, and Supplemental Table 3.

### SMuRF-less AMI in the population with overweight/obesity

In Singapore, the projected prevalence of SMuRF-less AMI in the overall AMI population with overweight/obesity is forecasted to rise from 2.5% (470 of 18,523; 95% CI: 1.8%-3.3%) in 2025 to 4.5% (1,999 of 44,515; 95% CI: 1.7%-7.3%) by 2040, with a CAGR of 3.9% (95% CI: 3.3%-4.5%) exceeding that of the total SMuRF-less AMI growth rate (CAGR 2.6%; 95% CI: 2.0%-3.2%). Similarly in the United Kingdom, the prevalence of SMuRF-less AMI with overweight/obesity will increase from 12.8% (6,851 of 53,659; 95% CI: 11.8%-13.7%) to 17.6% (9,196 of 52,172; 95% CI: 15.4%-19.8%), with a CAGR of 2.2% (95% CI: 1.4%-3.0%) exceeding that of the total SMuRF-less AMI (CAGR 1.3%; 95% CI: 0.5%-2.1%) (Figure 2, Supplemental Table 2, Supplemental Figure 8). The projected case fatality rate of the SMuRF-less AMI population with overweight/obesity, as well as sex- and age-specific subgroup analysis, can be found in Figure 2, Supplemental Table 3 and Supplemental Figures 8 to 10.

## Discussion

Although previous studies have focused on the upward trajectory of the prevalence of AMI and the contribution of metabolic burden,^3^^,^^23^^,^^24^ little is known about the future of the often-overlooked SMuRF-less population presenting with AMI. This is particularly important given reports of the increased proportion of AMI patients with no SMuRFs over the last decade, the higher rates of early mortality in this population, and the lack of any strategy to prevent recurrent cardiovascular events in this group. This multinational, population-based forecast study adds to the present knowledge with several key findings:

1. From 2025 to 2040, the prevalence of AMI in the SMuRF-less population is anticipated to show year-on-year growth of 2.6% and 1.3% across both Singapore and the United Kingdom, respectively, and contribute to a much larger proportion of the overall AMI census. The proportion of SMuRF-less AMI mortality of total AMI mortality is set to decrease in the United Kingdom but increase in proportion in Singapore.
2. Several phenotypic differences can be observed in the population with SMuRF-less AMI. Namely, the sex disparity in SMuRF-less AMI is predicted to widen further, with men bearing majority of the total SMuRF-less AMI prevalence. The age group most vulnerable to SMuRF-less AMI will also shift toward a younger cohort. SMuRF-less STEMI presentations will continue to carry the highest case fatality rate among all SMuRF-less AMI by 2040.
3. The rise in AMI prevalence is anticipated to be more rapid in the SMuRF-less population with overweight/obesity, compared with that of the overall SMuRF-less population, making up a greater proportion of total AMI cases by 2040. The majority of SMuRF-less AMI prevalence with overweight/obesity will predominantly affect middle-aged adult men.

In the years to come, Singapore and the UK health care systems will face unique sets of challenges in the prevention and management of SMuRF-less AMI. In Singapore, health care systems will see a rapid increase in SMuRF-less AMI prevalence, raising the need for expanding preventative strategies to the SMuRF-less population at risk of coronary artery disease.^25^ Singapore’s lower prevalence of SMuRF-less AMI compared with the Western counterparts in the past decade,^10^ is largely caused by the higher metabolic burden reported in the Asian population and likely competing risks.^23^^,^^26^^,^^27^ Current population-based primary prevention programs, such as the Healthier SG initiative, that offer nationwide screening of atherosclerotic CVD risk based on the presence of SMuRFs, may inadvertently overlook the vulnerable SMuRF-less population with coronary artery disease, leading to less aggressive risk factor management in individuals who do not meet the SMuRF criteria.^3^^,^^28^ On the other hand, the United Kingdom will bear a higher burden of SMuRF-less AMI prevalence, with its proportion of SMuRF-less individuals in the overall AMI cohort doubling that of Singapore by 2040. Despite reductions in the prevalence of SMuRFs in the UK population,^29^ the relatively high and growing burden of SMuRF-less AMI in the United Kingdom is of concern. As such, clinicians should be cognizant in the detection and management of nontraditional modifiable atherosclerotic risk factors that include, but are not limited to, deleterious behavioral habits,^30^ metabolic dysfunction-associated steatotic liver disease,^31^, ^32^, ^33^ systemic inflammatory conditions,^11^^,^^26^^,^^34^ and high lipoprotein(a) concentrations.^35^, ^36^, ^37^, ^38^, ^39^, ^40^, ^41^, ^42^, ^43^, ^44^, ^45^, ^46^

The SMuRF-less AMI case fatality rate is projected to decrease in both the United Kingdom and Singapore. This is attributed to the improving prescription rates of guideline-directed secondary preventative medical therapy and lifestyle modifications.^47^^,^^48^ However, it is important to note that the proportion of SMuRF-less mortality in the total AMI mortality in Singapore is rising.^7^^,^^9^ We, and others,^7^^,^^9^ hypothesize that ventricular arrhythmias may be the key drivers of early excess mortality following SMuRF-less AMI, given the increased rates of cardiac arrest,^7^^,^^16^ as well as left main and left anterior descending coronary artery involvements, which portend larger territories of myocardial infarction.^10^^,^^49^, ^50^, ^51^

As a reflection of the sex-specific trends of overall AMI worldwide,^2^^,^^24^ men will continue to bear a disproportionate burden of SMuRF-less AMI cases in Singapore and the United Kingdom. This sex-based disparity in SMuRF-less AMI prevalence is underpinned by a multitude of factors related to the protective effect of estrogen on women’s cardiovascular health, as well as deleterious male-dominant behavioral factors that include excessive alcohol consumption and poor dietary habits.^52^ Although men will continue to bear the majority of SMuRF-less AMI cases, the study emphasizes that shining the spotlight on women’s cardiovascular health remains a priority in the field of SMuRF-less AMI.^53^ Similar to the SWEDEHEART registry that demonstrated higher 30-day all-cause mortality rates in SMuRF-less women compared with men following STEMI,^9^ our forecast analysis showed that SMuRF-less AMI case fatality rates will remain higher in women than in men from 2025 to 2040 in the UK population, but not in the Singapore cohort. The worse prognostic outcomes observed in SMuRF-less women have been described to be multifactorial, attributing to differences in pathophysiology (ie, women tended to have less extensive coronary disease and myocardial ischemic preconditioning, leading to increased vulnerability to ischemia), and quality of care (ie, delayed STEMI presentations in women^4^^,^^10^^,^^11^^,^^16^^,^^53^^,^^54^). Future studies are needed to examine the biological and genetic variations in Eastern and Western SMuRF-less populations, contributing to the excess case fatality rates in SMuRF-less women observed in the United Kingdom, but not in Singapore.

Furthermore, the shift of SMuRF-less AMI burden to the working-age demographic by 2040 will likely be characterized by an increase in health care usage, loss in productivity, and/or premature death.^55^ In the past 2 decades, the decline in overall AMI prevalence in the United Kingdom has been largely restricted to those aged above 60 years, with no improvements in younger adults.^29^ This is most likely contributed by the ever-increasing rates of overweight/obesity and subclinical metabolic diseases in younger and middle-aged adults, attributed to sedentary living and easier access to high-calorie, low-nutrient-dense foods.^56^, ^57^, ^58^ In contrast, older populations with AMI are primarily driven by SMuRFs, allowing for easier detection and treatment of risk factors.^3^ As such, current atherosclerotic CVD risk stratification systems may inadvertently overlook the younger vulnerable population given that these tools prioritize the presence of SMuRFs and older age.^29^

Obesity was not included as part of the SMuRFs in the harmonized definition of SMuRF-less coronary artery disease.^11^ Studies have reported lower BMI in people with SMuRF-less AMI than their counterparts with SMuRFs, alluding to the notion that obesity may not be the key driver of advanced atherosclerosis^10^ in the SMuRF-less population. However, our present forecast analysis challenges this hypothesis and indicates that SMuRF-less AMI prevalence in individuals with overweight/obesity will increase, with a higher proportion of SMuRF-less AMI with overweight/obesity in the United Kingdom, more than 4 times higher than that predicted in Singapore. Although there is mixed evidence for diet-based weight loss,^59^ the emergence of newer glucose-lowering therapies such as glucagon-like peptide-1 receptor agonists on weight loss and cardiometabolic outcomes^60^ may shift evidence-based clinical practice in SMuRF-less coronary disease^11^ with overweight/obesity.^5^^,^^61^, ^62^, ^63^, ^64^, ^65^, ^66^, ^67^ As such, overweight/obesity is a modifiable factor that should be addressed in the clinical pathway of SMuRF-less coronary artery disease.^11^

### Study limitations

First, the current study uses Poisson model for projection of our data which runs the risk of overdispersion and assumes a linear relationship between the logarithm of frequency or rate. Hence, it may not fully account for potential interactions between nontraditional risk factors, such as obesity, that may follow nonlinear patterns caused by saturation effects or interventions.^4^ This also assumes that trends observed from the years 2010 to 2018 will remain consistent and continue in the future without fully accounting for the subtle and complex phenomena involving dynamic changes in medico-pharmaceutical care, improvements in risk factor treatment, and the impacts of climate change or warfare, that may alter disease trajectories in ways that cannot be accurately predicted. Moreover, the Poisson regression models were not adjusted for individual-level covariates such as pharmacotherapy. As such, one must remain cognizant of overinterpreting marginally significant trends. Nevertheless, a time-series modelling framework was adopted, where the Poisson regression was fitted using time as the covariate. This approach enables the effects of evolving factors—such as pharmacotherapy uptake, comorbidities, and other temporal influences—to be indirectly captured through the historical patterns of the time series. Second, the current study uses BMI as a surrogate measure for overweight/obesity. Numerous studies have shown that the use of more anthropometric measurements (ie, waist-to-hip ratio) are better predictors of cardiovascular outcomes compared with BMI.^68^, ^69^, ^70^, ^71^, ^72^ Third, there was heterogeneity in the risk factor definitions across registries, potentially introducing systematic bias in the classification of SMuRF-less AMI and affecting cross-country comparisons. Moreover, the current strategy of categorizing patients into SMuRF and SMuRF-less based on binary thresholds is not ideal, as majority of these risk factors (eg, glycated hemoglobin A1c) share linear relationships with cardiovascular mortality. Thus, patients with borderline measurements of specific risk factors who do not yet meet the diagnostic threshold may be categorized as SMuRF-less, even though they are at elevated cardiovascular risk.^10^^,^^73^ Fourth, some stratified forecast estimates are based on small sample sizes of AMI cases that may inevitably inflate uncertainty. The subgroups with small sample sizes, particularly in SMuRF-less AMI in the young adult and STEMI subpopulations, may account for “unstable” forecasts that warrant cautious interpretation. Last, the COVID-19 pandemic should also be considered when interpreting the data projections, given the multiple associations between the pandemic and COVID-19 vaccinations with cardiovascular mortality and morbidity. However, the study did not project SMuRF-less AMI estimates using historical trends from 2019 to 2021 to avoid inaccuracies contributed by the initial volatile phase of the pandemic that saw sharp rises in global mortality despite previous decreasing trends.^74^

## Conclusions

From 2025 to 2040, the prevalence of SMuRF-less AMI will continue to rise in Singapore and UK populations with year-on-year growth of 2.6% and 1.3%, respectively, contributing to a larger proportion of the overall AMI census. The proportion of SMuRF-less mortality of total AMI mortality is set to decrease in the United Kingdom but will contribute to an increasing proportion of total AMI mortality in Singapore by 2040. Specific population groups vulnerable to SMuRF-less AMI will shift toward a younger, male-dominant demographic, with overweight/obesity as a major risk factor. These projections imply that health care policy needs to address nontraditional modifiable risk factors in CVD prevention and pay equal attention to men and women. The prioritization of overweight/obesity management, in addition to the 4 SMuRFs, requires urgent attention.

### Data Availability Statement

All data and code used in the analysis are available upon request to the corresponding author.

## Funding Support and Author Disclosures

This research was supported by the CArdiovascular DiseasE National Collaborative Enterprise (CADENCE) and the National Medical Research Council Research Transition Award (TA24jul-0008). Dr Chong receives research support from the Clinician Scientist Development Unit, Yong Loo Lin School of Medicine, National University of Singapore. Dr Hausenloy has received consultant fees from Faraday Pharmaceuticals Inc and Boehringer Ingelheim International GmbH; has received honoraria from Servier; has received research funding from AstraZeneca, Merck Sharp and Dohme Corp, and Novo Nordisk; and is supported by the Duke-NUS Signature Research Programme funded by the Ministry of Health, Singapore Ministry of Health’s National Medical Research Council under its Singapore Translational Research Investigator Award (MOH-STaR21jun-0003), Centre Grant scheme (NMRC CG21APR1006), and Collaborative Centre Grant scheme (NMRC/CG21APRC006). Dr Richards holds the New Zealand Heart Foundation Chair of Cardiovascular Studies; and has received advisory board fees and/or grant support from Roche Diagnostics, Novo Nordisk, Abbott Laboratories, Thermo Fisher, Medtronic, Sphingotec, Novartis, and AstraZeneca. Dr Chan receives speaker’s fees and research grants from AstraZeneca, Abbott Technologies, and Boston Scientific; and receives salary support from a National Medical Research Council Clinician Scientist Award-Senior Category (MOH-000280-00). Dr Chew has received research grant support from NUHS Seed Fund (NUHSRO/2022/RO5+6/Seed-Mar/03), National Medical Research Council Research Training Fellowship (MH 095:003/008-303), National University of Singapore Yong Loo Lin School of Medicine's Academic Fellowship Scheme, the NUHS Clinician Scientist Program (NCSP2.0/2024/NUHS/NCWS), CSDU Clinician-Scientist Grant, and the National Medical Research Council Research Transition Award (TA24jul-0008). All other authors have reported that they have no relationships relevant to the contents of this paper to disclose.
