# Supplementary material for: Myocardial Infarction Without Standard Modifiable Risk Factors From 2025-2040: Forecast Analysis of Multinational, Population-Based Study
Source: JACC Asia. 2026 Mar 20;6(6):910–25. doi: 10.1016/j.jacasi.2026.01.027 (PMC13244099; doi:10.1016/j.jacasi.2026.01.027)
Supplement: Supplemental Material [file mmc1.docx]

**Appendix (Supplemental Materials)**

**Myocardial Infarction without Standard Modifiable Risk Factors from 2025-2040: Forecast Analysis of Multinational, Population-Based Study**

**CONTENTS**

|  | **Page** |
| --- | --- |
| **Supplemental Material 1:** Sensitivity analysis for ICD 10 coding transition | 4 |
| **Supplemental Material 2:** Definition of standard modifiable risk factors | 6 |
| **Supplemental Material 3:** Sensitivity analysis for model stability with inclusion of 2019 data | 7 |
| **Supplemental Figure 1:** Multiple Imputation output, with number of imputed observations, covariates and output, distribution of BMI across imputed datasets | 8 |
| **Supplemental Material 4:** Sensitivity analysis of the United Kingdom imputed dataset | 12 |
| **Supplemental Material 5:** Goodness-of-fit for Poisson regression models | 14 |
| **Supplemental Material 6:** Assessment of regression model performance | 16 |
| **Supplemental Material 7:** Comparative forecasting approaches | 18 |
| **Supplemental Table 1:** Proportion of SMuRF-less AMI out of total AMI from 2025 to 2040. | 20 |
| **Supplemental Table 2:** Projected SMuRF-less AMI prevalence from 2025 to 2040, stratified by sex and age category. | 21 |
| **Supplemental Figure 2:** Projected total AMI prevalence in Singapore and UK from 2025 to 2040. Bar charts depict crude AMI cases and line graphs depict AMI prevalence. | 23 |
| **Supplemental Figure 3:** Proportion of projected SMuRF-less AMI prevalence and mortality out of total AMI in Singapore and UK from 2025 to 2040 | 24 |
| **Supplemental Table 3:** Projected SMuRF-less AMI case fatality rate from 2025 to 2040, stratified by sex and age category | 25 |
| **Supplemental Figure 4:** Projected SMuRF-less AMI case fatality rate from 2025 to 2040, stratified by (A) Male and, (B) Female. Bar charts depict crude AMI mortality and line graphs depict case fatality rate of AMI. | 27 |
| **Supplemental Figure 5:** Proportion of projected SMuRF-less AMI cases from 2025 to 2040, stratified by age category in (A) Singapore and, (B) UK. | 28 |
| **Supplemental Figure 6:** Projected SMuRF-less AMI case fatality rate from 2025 to 2040, stratified by (A) Middle-aged adults and, (B) Older adults. Bar charts depict crude AMI mortality and line graphs depict case fatality rate of AMI. | 29 |
| **Supplemental Figure 7:** Projected SMuRF-less AMI case fatality rate from 2025 to 2040, stratified by (A) STEMI and, (B) NSTEMI. Bar charts depict crude AMI mortality and line graphs depict case fatality rate of AMI. | 30 |
| **Supplemental Figure 8:** (A) Projected SMuRF-less AMI prevalence and, (B) Projected SMuRF-less AMI case fatality rate in overweight/obesity population from 2025 to 2040. Bar charts depict crude SMuRF-less overweight/obesity AMI cases or mortality and line graphs depict SMuRF-less overweight/obesity AMI prevalence or case fatality rate | 31 |
| **Supplemental Figure 9:** (A) Proportion of projected SMuRF-less AMI cases and, (B) Proportion of projected SMuRF-less AMI mortality in overweight/obesity population in Singapore and UK from 2025 to 2040, stratified by sex | 32 |
| **Supplemental Figure 10:** Proportion of projected SMuRF-less AMI cases in overweight/obesity population from 2025 to 2040, stratified by age category in (A) Singapore and, (B) UK | 33 |

**
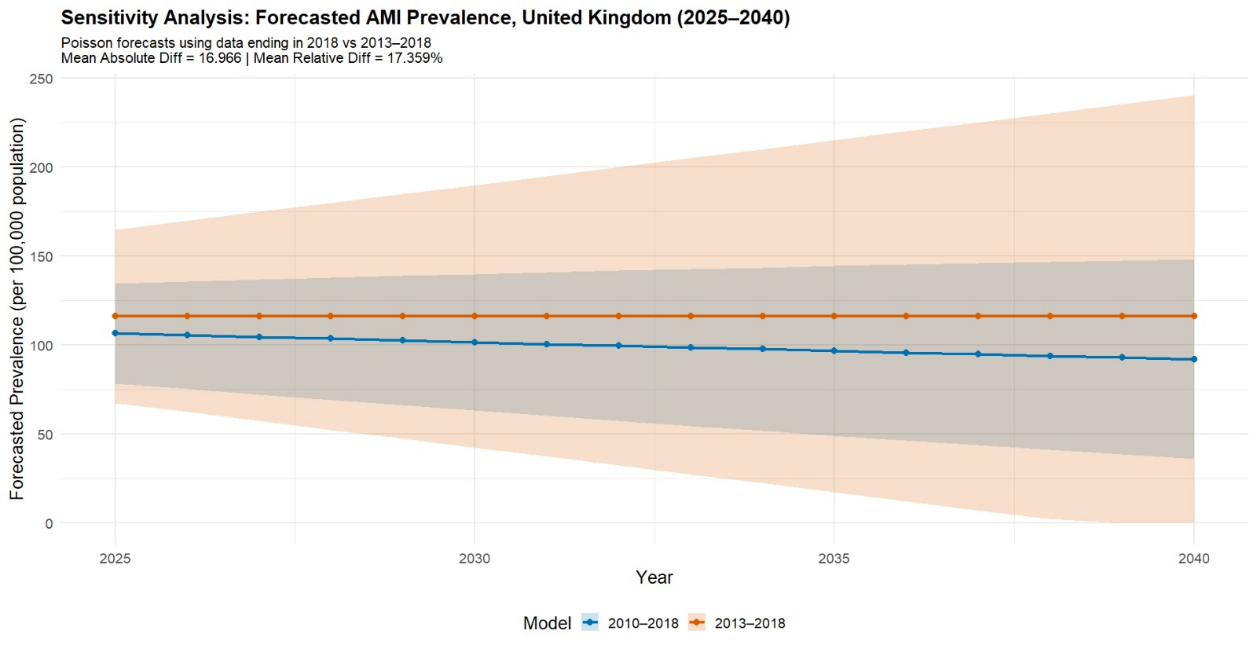

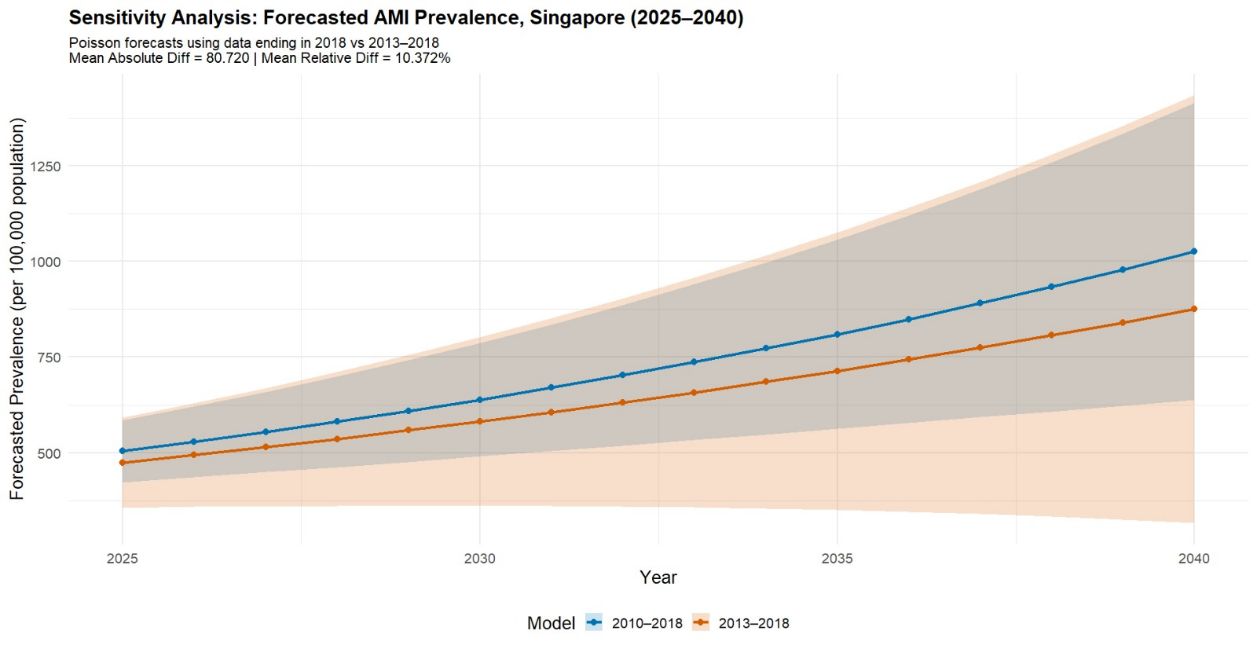
Supplemental Material 1: Sensitivity analysis for ICD 10 coding transition**

To evaluate whether the 2012 transition from ICD-9 to ICD-10 coding introduced any discontinuity in AMI case ascertainment, we conducted sensitivity analyses by refitting the Poisson regression models using two alternative observation windows:

- Model A: 2010–2018 (baseline, spanning both ICD-9 and ICD-10 periods)
- Model B: 2013–2018 (post-ICD-10 period only)

Both models were applied to forecast AMI prevalence for 2025–2040, with 95% confidence intervals (CIs) derived using the Delta method. The analyses were performed separately for Singapore and the United Kingdom, where the coding transition occurred in comparable timeframes (2011–2012).

For Singapore, the two models produced highly consistent trajectories, with substantial overlap of the 95% CIs across the projection horizon. The mean absolute forecast difference between the models was 80.7 cases per 100,000 population, corresponding to a mean relative difference of 10.4%—well within the uncertainty bounds of both models.

For the United Kingdom, forecast trajectories were similarly stable, with a mean absolute difference of 17.0 cases per 100,000 and a mean relative difference of 17.4%. The 95% CIs exhibited strong concordance, indicating that the post-ICD-10 period model captured equivalent underlying trends.

These findings suggest that the 2012 ICD-9 to ICD-10 coding switch did not materially affect the continuity or stability of AMI case ascertainment in either dataset. The consistency of forecasts across model specifications supports the robustness of the Poisson regression framework and confirms that no artificial breakpoint was introduced by the coding transition.

**Supplemental Material 2: Definition of standard modifiable risk factors.**

In the Singapore Myocardial Infarction Registry (SMIR), the presence of type 2 diabetes mellitus (T2DM) was defined by either a) fasting blood glucose ≥7.0 mmol/L or random glucose ≥11.1 mmol/L during the index admission, b) a past diagnosis of T2DM, or c) the consumption of glucose-lowering medications^1, 2^. Hypertension was based on a) systolic blood pressure >130 mmHg and/or diastolic blood pressure >85 mmHg during the index admission, b) a past diagnosis of hypertension, or 3) the background use of anti-hypertensive medications ^3, 4^. Hyperlipidemia was defined by 1) total cholesterol >6.2 mmol/L, low-density lipoprotein cholesterol >4.1 mmol/L, or triglyceride >1.7 mmol/L, 2) previous diagnosis of hyperlipidemia, or 3) on background lipid-lowering therapy ^1, 5, 6^. In the Myocardial Ischemia National Audit Project (MINAP), hyperlipidemia was recorded as any patient with elevated lipids recorded at admission, or already taking lipid-lowering therapy. T2DM status was defined by a current diagnosis of T2DM from the community, or receiving dietary, pharmacotherapy, or insulin therapy. Hypertension status was based on either two sustained values >140/90 mmHg prior to admission, or currently undertaking lifestyle measures or blood pressure-lowering medical therapy^7, 8^. For both SMIR and MINAP, smoking included both active and previous smokers.

Given the missing data for body mass index (BMI) in MINAP, multiple imputations with chained equations (MICE) were used to impute values for BMI. MICE is the best practice when dealing with missing data and can provide unbiased estimates even when levels of missing data are significant, and some protection when the pattern of ‘missingness’ are not at random^9^. We created ten imputed datasets and extracted a single dataset for presentation of trends of patients with overweight/obesity in MINAP.

**Supplemental Material 3: Sensitivity analysis for model stability with inclusion of 2019 data
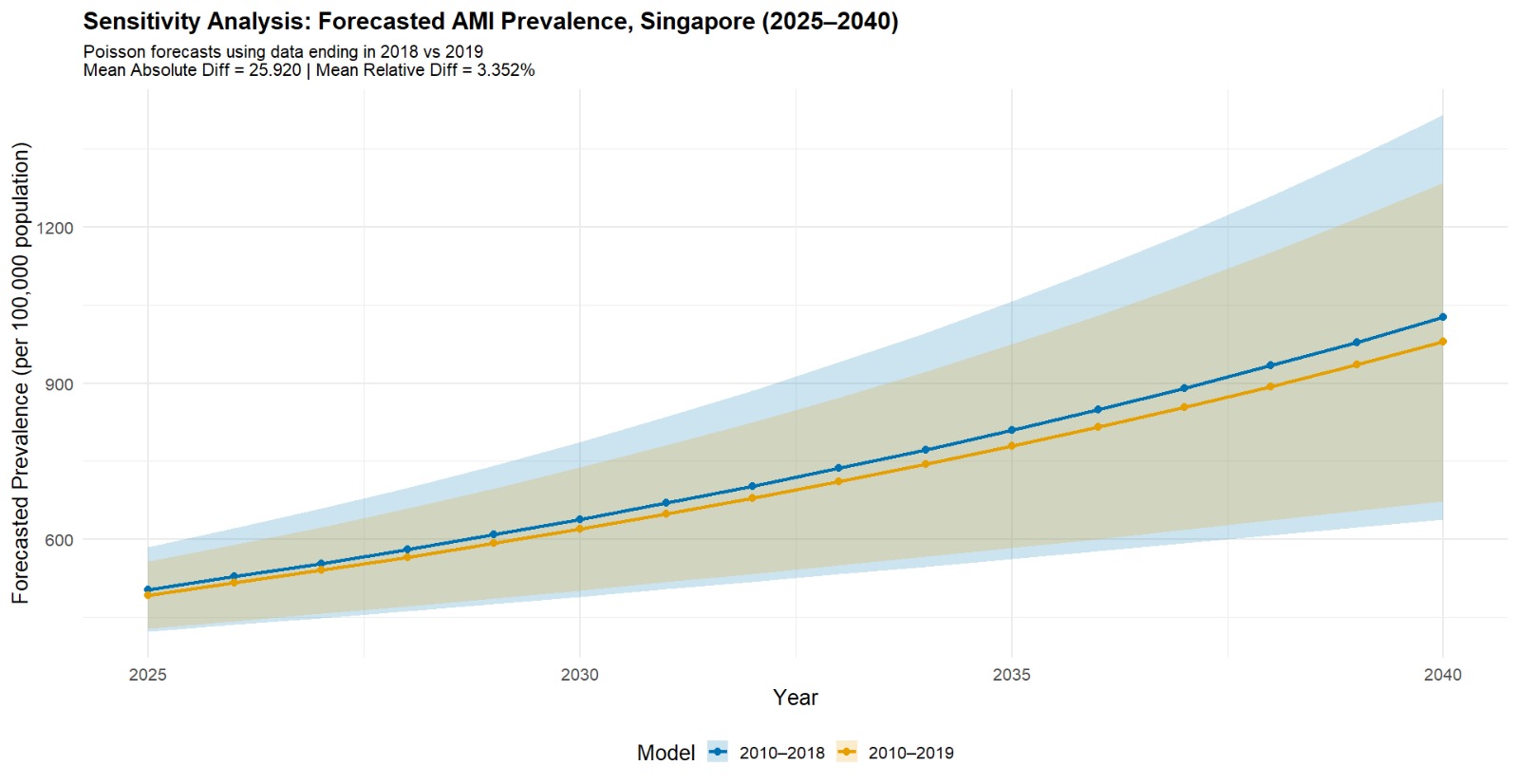
**

The exclusion of 2019-2021 data was to avoid pandemic-related distortions of the long-term projections. To assess whether exclusion of year 2019 (i.e. COVID-19–affected years) biased long-term projections, we refitted two Poisson regression models using data from 2010-2018 (baseline) and 2010-2019, and both were used to forecast AMI crude prevalence rates for 2025–2040 with 95% confidence intervals. Forecast trajectories were compared against the baseline (2010–2018) model, and the mean absolute and relative forecast differences were computed. The resulting forecast trajectories were similar, with a mean absolute difference of 25.9 and a mean relative difference of only 3.35% across the projection period. The 95% confidence intervals substantially overlapped, indicating that inclusion of 2019 data did not affect the estimated trend or its uncertainty range. Consistent forecast trajectories across all models demonstrated that the inclusion of 2019 data did not affect long-term projections. The Poisson model therefore remains stable and robust to the inclusion of pre-pandemic and early-pandemic years.

**Supplemental Figure 1: Multiple Imputation output, with number of imputed observations, covariates and output; distribution of BMI across imputed datasets**

Given the significant proportion of missing data for BMI in the remaining patient records, the study analysis imputed BMI as a monotone variable alone. We created ten imputed datasets in our initial imputation model. Although we used an imputed model that would typically undertake multiple imputation by chained equations (MICE), the missing-value pattern was monotone. As such, additional iterations were not performed, and monotone imputation was used. The auxiliary variables included in the imputation model can be found below, alongside the output from the first imputed dataset.


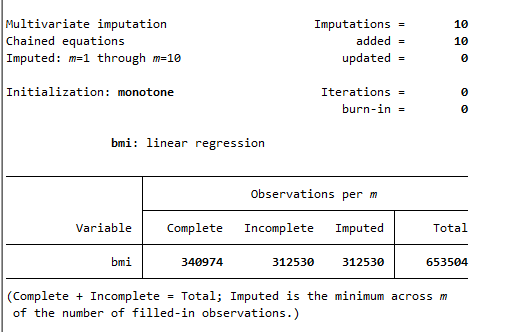


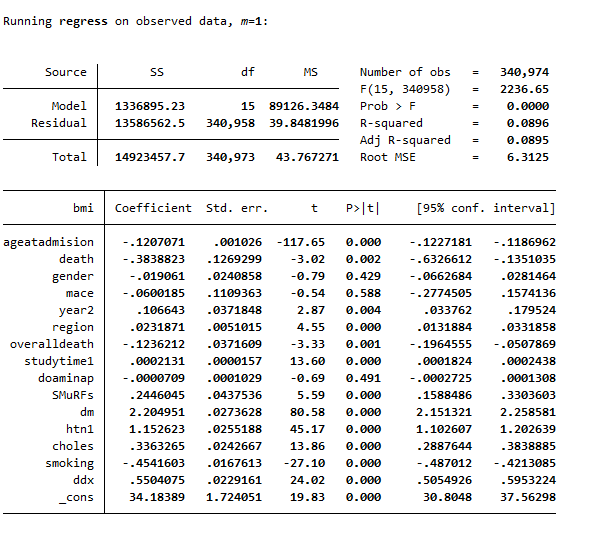


*Distribution of BMI across 10 imputed datasets (Kg/m^2^)*


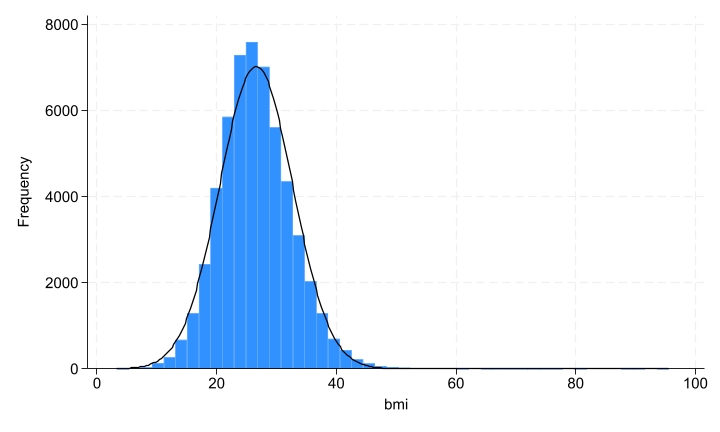


*MACE; Major adverse cardiovascular events. “Year2” refers to year of admission with AMI. “Doaminap” is date of admission with AMI. “Ddx” is diagnosis, a categorical variable of either NSTEMI or STEMI. SMuRFs; Standard modifiable cardiovascular risk factors. “Dm” is diabetes mellitus. “Htn1” is hypertension, “Choles” is hypercholesterolemia.*

Although the pooling results across imputations using Rubin’s rules is best practice, especially when using the resulting BMI variable in analytical models, this presented difficulties for our study design. Data supplied from MINAP were prevalence and mortality rates per 100,000 of the England and Wales population, therefore in order to present numbers reflective of the England and Wales population size, we needed to use a single imputed dataset, rather than the total pooled patient number across 10 imputed datasets. Distribution of the resulting BMI across imputed datasets was checked, alongside examining of median BMI with IQR, with results being similar; thus ensuring there is not significant variability in the generated BMI across imputations.

Multiple imputation was performed under the MAR (Missing at Random) assumption, whereby missingness may depend on observed variables included in the imputation model. This assumption was supported by exploratory analyses showing that BMI missingness was associated with observed factors such as calendar time and mortality status. To enhance transparency, we provide a comprehensive missing data assessment summarizing the extent of missingness across key variables and identifying those included in the imputation model.

The table below presents the percentage of missing overweight AMI prevalence in the UK dataset, overall and stratified by sex and age group. A clear temporal pattern is observed, with higher levels of missingness in earlier calendar years (2010–2012) followed by a progressive reduction in missingness toward 2018. This pattern is consistent with improvements in data completeness over time.

Missingness patterns were broadly similar across sex and age strata, with no marked differential missingness between sex or across age groups. These findings suggest that missingness was primarily associated with calendar time rather than specific demographic subgroups, supporting the plausibility of the Missing at Random (MAR) assumption underlying the multiple imputation procedure.

| Year | Missing Percentage % | | | | | |
| --- | --- | --- | --- | --- | --- | --- |
|  | Overweight  Total | Overweight  Male | Overweight  Female | Overweight  Young adults | Overweight  Middle-aged Adult | Overweight  Older Adult |
| 2010 | 55.28 | 52.46 | 61.07 | 54.60 | 46.62 | 59.10 |
| 2011 | 51.68 | 48.60 | 58.15 | 53.59 | 41.52 | 55.93 |
| 2012 | 46.11 | 43.69 | 51.56 | 45.61 | 37.88 | 49.41 |
| 2013 | 41.59 | 41.84 | 49.91 | 43.61 | 35.32 | 48.14 |
| 2014 | 42.03 | 40.03 | 46.61 | 44.82 | 34.98 | 44.58 |
| 2015 | 40.69 | 38.82 | 45.05 | 46.82 | 34.14 | 42.84 |
| 2016 | 36.86 | 34.89 | 41.31 | 37.77 | 30.09 | 39.04 |
| 2017 | 33.95 | 32.07 | 38.14 | 37.42 | 27.70 | 35.75 |
| 2018 | 32.76 | 31.04 | 36.60 | 36.84 | 26.39 | 34.42 |

**Supplemental Material 4: Sensitivity analysis of the United Kingdom imputed dataset**

Missing body mass index (BMI) values in the United Kingdom dataset were addressed using multiple imputation by chained equations (MICE), which generated 10 imputed datasets. Within each imputed dataset, BMI was used to classify individuals into overweight/obesity and non-overweight/obesity groups. Annual time series were then constructed by counting the number of AMI cases with overweight/obesity from 2010 to 2018.

For each imputed dataset, Poisson regression models were fitted independently to the corresponding time series and used to generate forecasts for the period 2025 to 2040, together with 95% confidence intervals. Forecast estimates and confidence intervals were compared across all 10 imputed datasets to assess robustness to imputation uncertainty.

Across all imputed datasets, forecast trajectories showed consistent direction and magnitude, with substantial overlap of the 95% confidence intervals throughout the projection period. This consistency indicates that the MICE procedure produced stable time-series classifications and that the variables included in the imputation model adequately supported the imputation of BMI used for stratification.


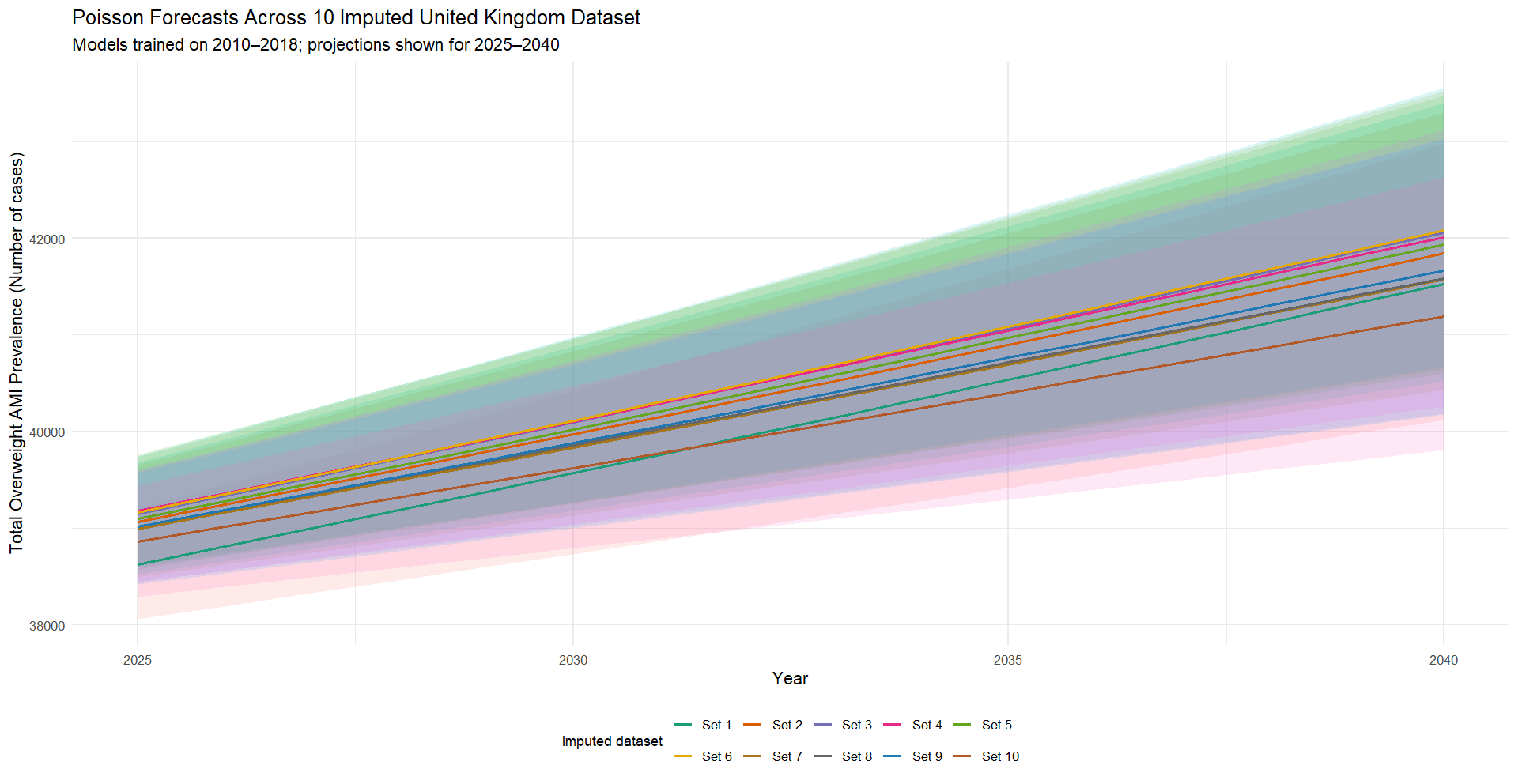


As an additional sensitivity analysis, we performed Poisson regression forecasting using complete-case data only, excluding individuals with missing BMI values and without applying imputation. Forecasts based on complete-case data (trained on 2010–2018 and projected to 2025–2040) showed a directionally consistent increasing trend compared with forecasts derived from the imputed datasets. As expected, absolute case counts were lower in the complete-case analysis due to the reduced sample size and lack of population representativeness; however, the temporal trend and direction of change were consistent.


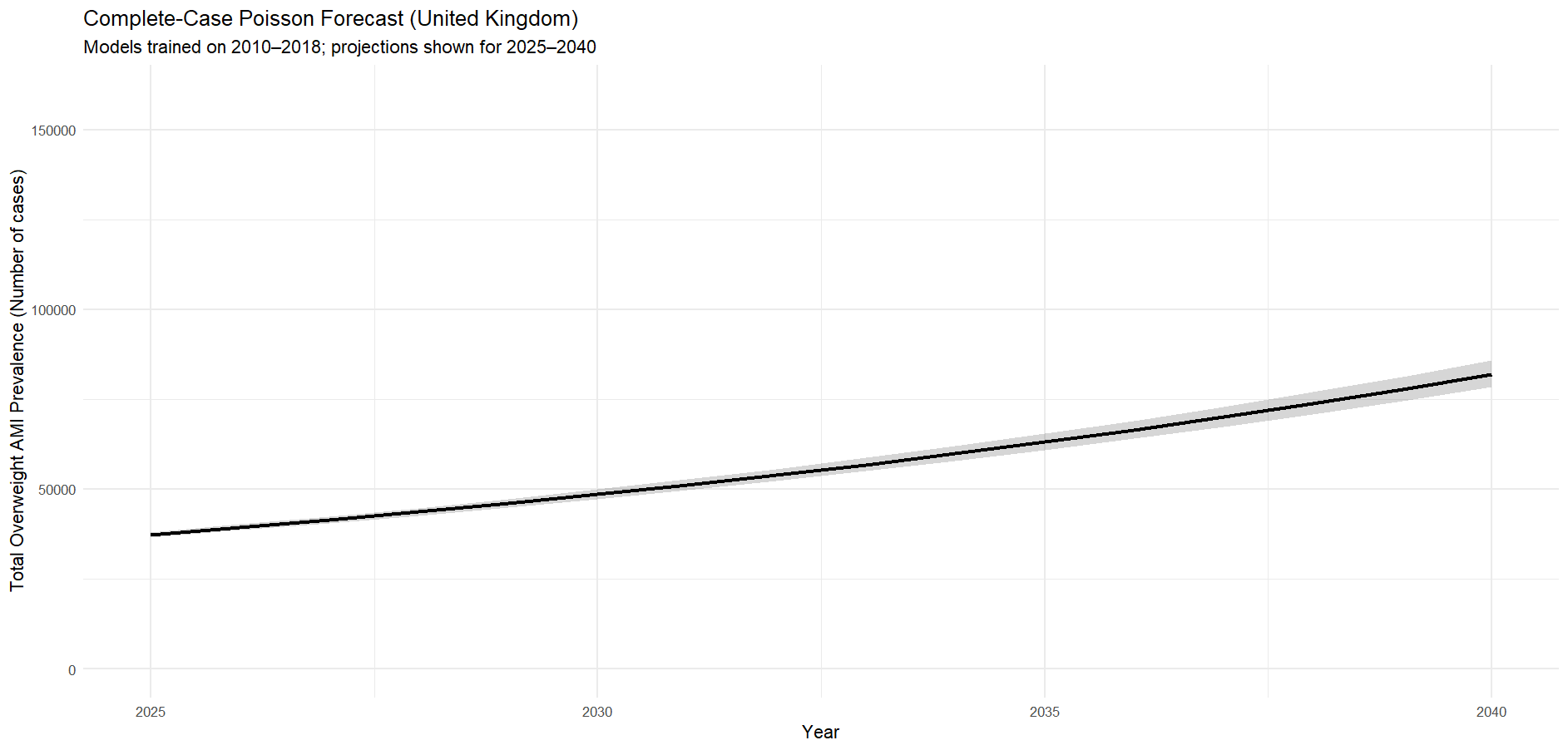


Taken together, the agreement across imputations and the concordance in forecast direction between imputed and complete-case analyses indicate that the primary conclusions are robust to the handling of missing BMI data and are not driven by the imputation procedure.

Given this consistency, a single representative imputed dataset was used for the primary forecasting analyses to simplify presentation. Thus, BMI was not included as a covariate in the Poisson regression models and did not directly influence model estimation beyond its role in stratifying the time-series into AMI groups with and without overweight/obesity.

**Supplemental Material 5: Goodness-of-fit for Poisson regression models**

To assess the performance of the model, internal validation was conducted involving extensive testing using the Deviance and Pearson Goodness-of-Fit tests, ensuring that the Poisson models adequately represented the historical datasets from 2010 to 2018. The Deviance test evaluates the model by comparing the likelihood of the fitted model against a saturated model, while the Pearson Goodness-of-Fit test measures the discrepancy between observed and expected frequencies. Specifically, the Pearson chi-square statistic is given by:

$$\chi^{2}= \sum_{i=1}^{n}\frac{{(O_{i}-E_{i})}^{2}}{E_{i}}$$

Where:

Oi = observed frequency for the i-th category.

Ei​ = expected frequency under the model for the i-th category.

The deviance statistic is computed as:

$$D=2\sum_{i=1}^{n}\left[ O_{i}\log(\frac{O_{i}}{E_{i}} \right)-(O_{i}-E_{i})]$$

Both statistics are approximately chi-square distributed with n – k − 1 degrees of freedom, where n is the number of observations and k is the number of estimated parameters. A p-value greater than 0.05 suggests no significant lack of fit, indicating that the model adequately captures the data structure. High p-values observed in the forecast models indicated no evidence of lack-of-fit, thus confirming the robustness of our models.

|  | **Deviance** | |  | **Pearson** | |  |
| --- | --- | --- | --- | --- | --- | --- |
|  | **Goodness-of-fit** | **p-value** | **Dispersion** | **Goodness-of-fit** | **p-value** | **Dispersion** |
| **Singapore** |  |  |  |  |  |  |
| Total AMI Prevalence Rate | 2.459513 | p=0.9301147 | p=0.35136 | 2.466301 | p=0.9296132 | p=0.35233 |
| Total AMI Mortality Rate | 1.151342 | p=0.9920044 | p=0.16448 | 1.178413 | p=0.9914155 | p=0.16834 |
| SMuRF-less AMI Prevalence Rate | 0.3353368 | p=0.9998543 | p=0.04791 | 0.3385932 | p=0.9998495 | p=0.04837 |
| SMuRF-less AMI Mortality Rate | 0.2504404 | p=0.9999458 | p=0.03578 | 0.2545005 | p=0.9999427 | p=0.03636 |
| **UK** |  |  |  |  |  |  |
| Total AMI Prevalence Rate | 0.744778 | p=0.997967 | p=0.10640 | 0.743813 | p=0.997976 | p=0.10626 |
| Total AMI Mortality Rate | 0.037518 | p=0.999999 | p=0.00536 | 0.037631 | p=0.999999 | p=0.00538 |
| SMuRF-less AMI Prevalence Rate | 0.150910 | p=0.999999 | p=0.02156 | 0.150602 | p=0.999990 | p=0.02151 |
| SMuRF-less AMI Mortality Rate | 0.04771 | p=0.999999 | p=0.00682 | 0.04772 | p=0.999999 | p=0.00682 |

Abbreviations: AMI – acute myocardial infarction, SMuRF – standard modifiable risk factor, UK – United Kingdom

**Supplemental Material 6: Assessment of regression model performance**


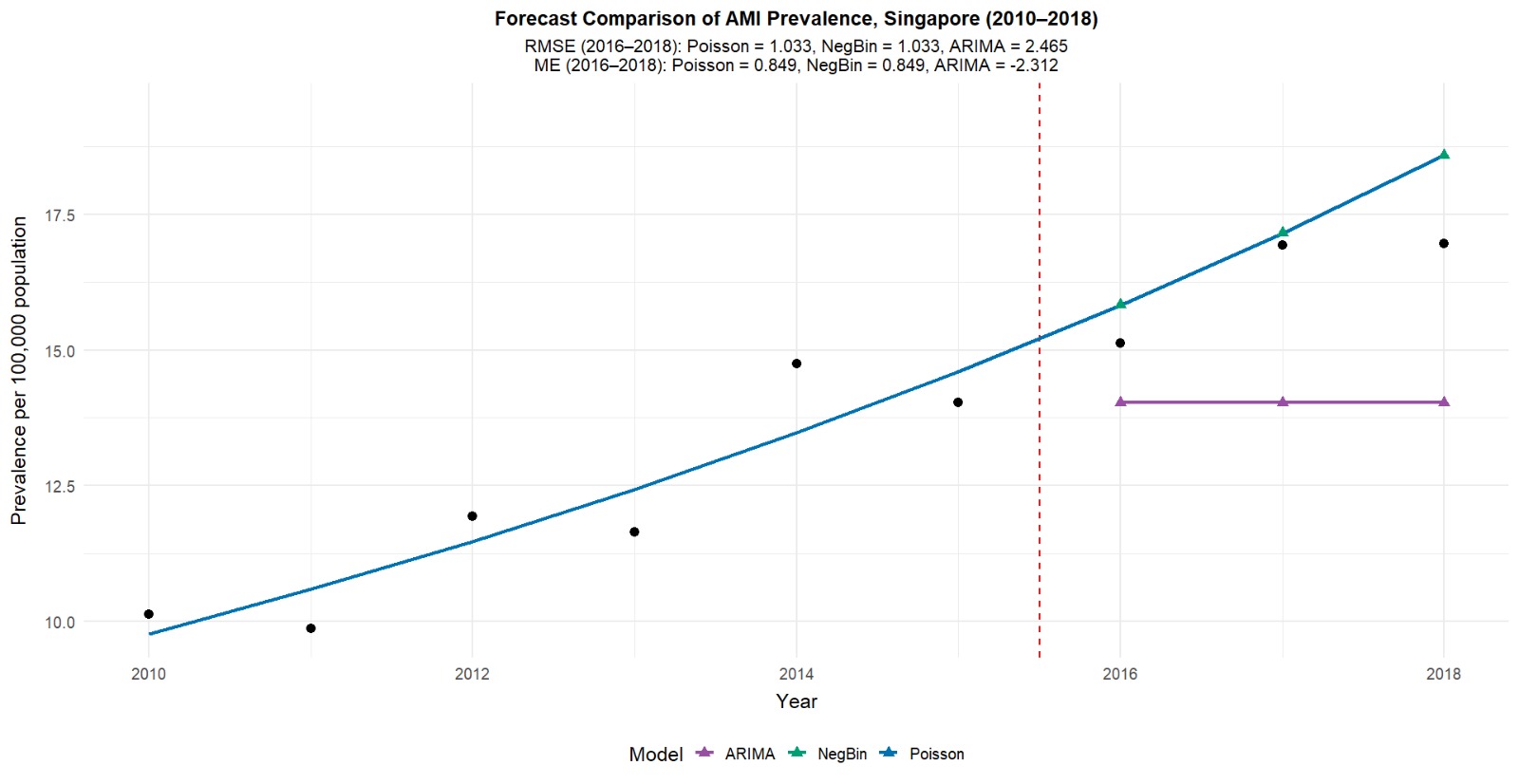
 The ARIMA model specification was selected by comparing multiple candidate parameterizations using the Akaike Information Criterion (AIC) and Bayesian Information Criterion (BIC), with the final model chosen based on optimal information-criterion fit. Autocorrelation (ACF) and partial autocorrelation (PACF) diagnostics were subsequently examined to inform the selection of autoregressive (p) and moving-average (q) terms. The order of differencing (d) was determined using unit-root–based heuristics, including the KPSS and Augmented Dickey–Fuller tests, to assess stationarity.

This figure compares the performance of the Poisson regression model against the Negative Binomial and Autoregressive Integrated Moving Average (ARIMA [0,1,0] with not drift/intercept term) models, each applied as time-series forecasting approaches for the SMIR and MINAP datasets. Internal validation was performed by training the models on historical data from 2010–2015 and generating out-of-sample forecasts for 2016–2018. Model performance was quantitatively assessed using Root Mean Square Error (RMSE) and Mean Error (ME), which measure predictive accuracy and bias, respectively.

The Poisson and Negative Binomial models demonstrated nearly identical forecast accuracy, with comparable RMSE and ME values—indicating minimal overdispersion and confirming that the simpler Poisson model provides an adequate fit to the data. In contrast, the ARIMA model exhibited substantially higher prediction errors, highlighting its poorer extrapolation capability for these epidemiological count trends. Overall, these results support the use of the Poisson regression model as a robust and parsimonious framework for projecting future AMI prevalence and mortality trends (2025–2040).

**Supplemental Material 7: Comparative forecasting approaches
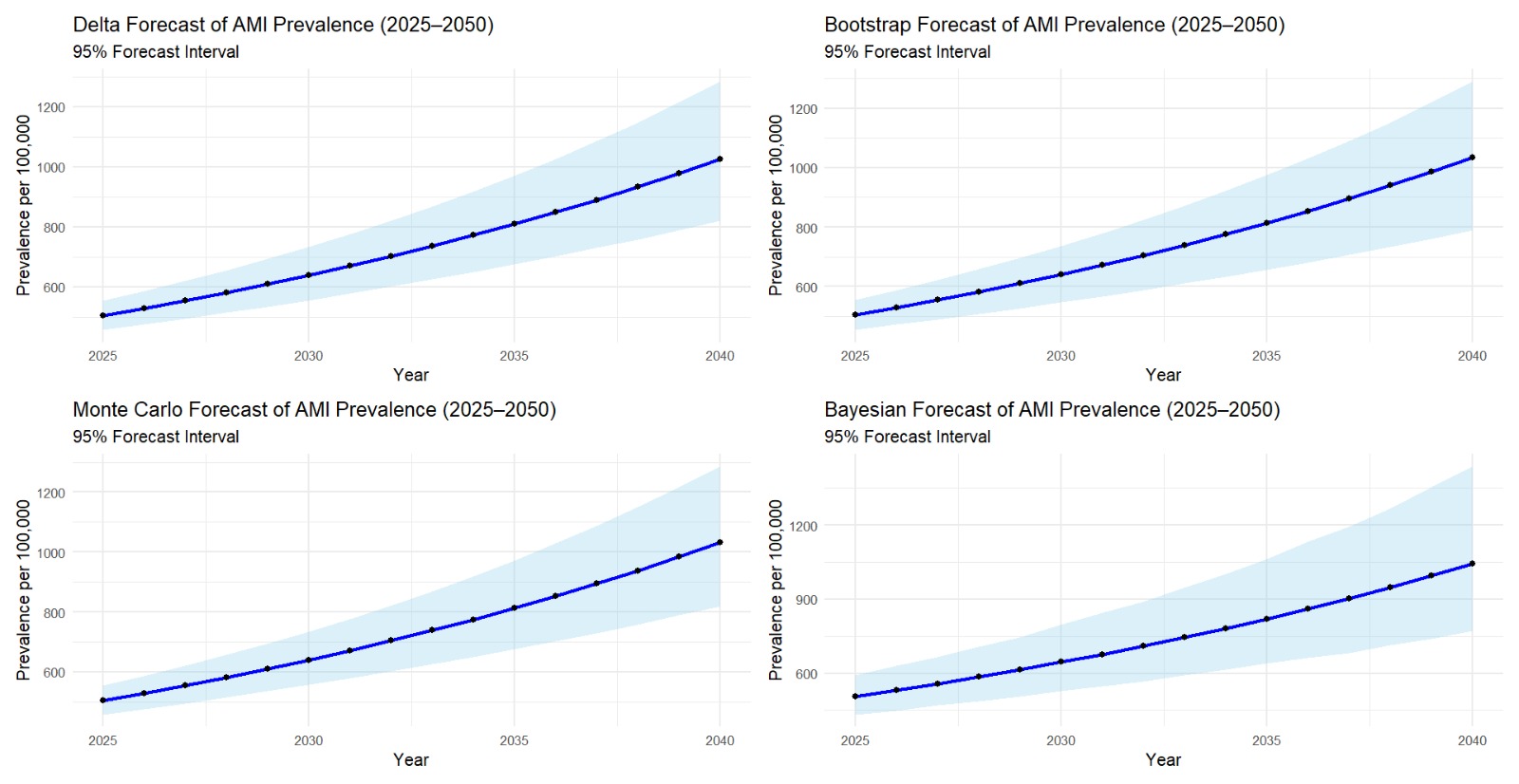
**
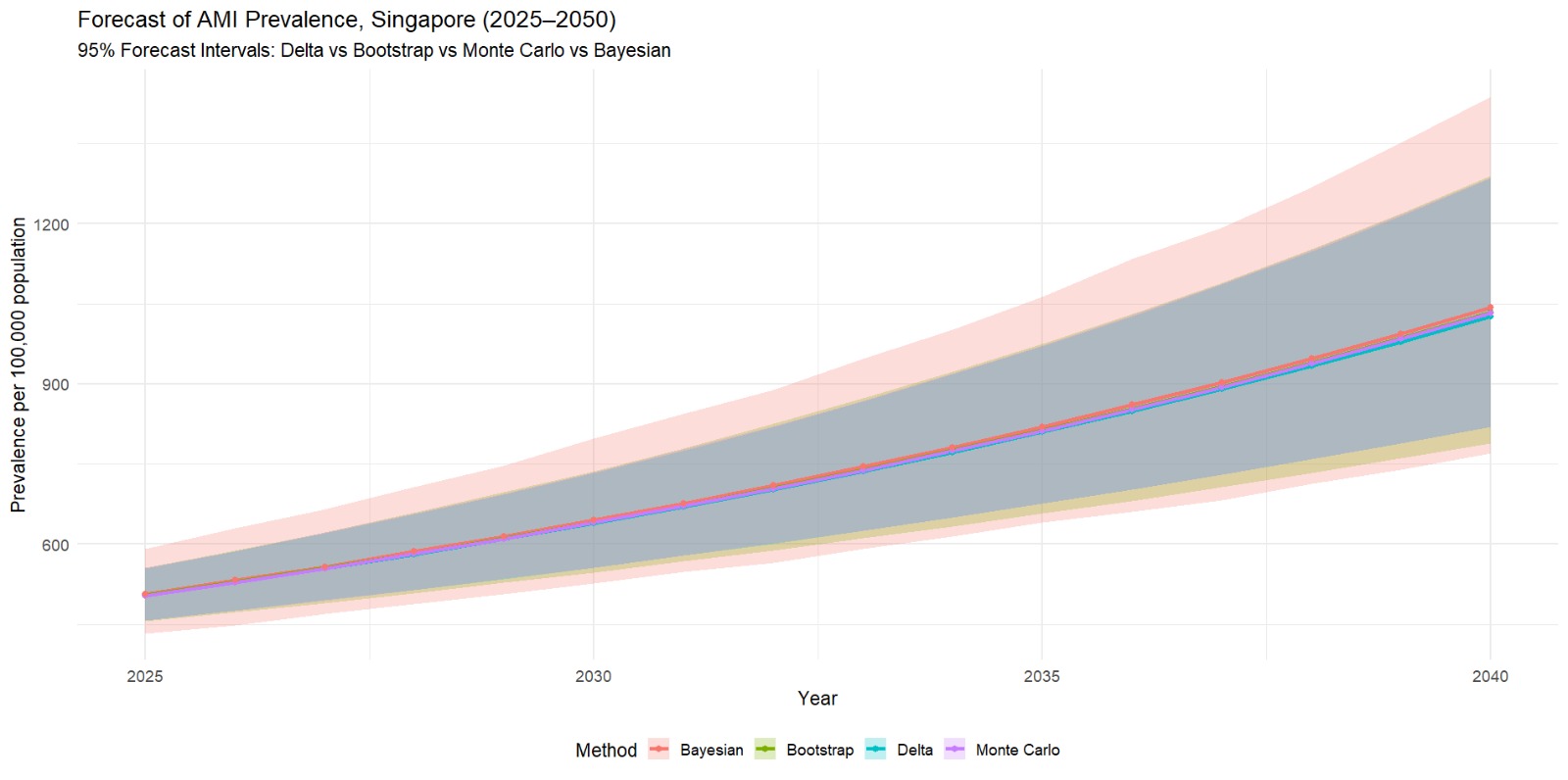


This figure compares four forecasting approaches—the Delta method, Bootstrap resampling, Monte Carlo simulation, and Bayesian posterior predictive estimation—applied to the projection of acute myocardial infarction crude prevalence rate in Singapore for the period 2025–2040. All forecasts were based on Poisson regression models fitted to historical national data (2010–2018) and projected under a consistent model specification to assess the uncertainty of long-term epidemiological trends.

The Delta method approximates forecast uncertainty analytically using the first-order Taylor expansion of the estimated model. The variance of each predicted rate is obtained from the model’s variance–covariance matrix of coefficients, assuming linear error propagation and fixed model parameters. This approach produces narrower 95% confidence intervals and is computationally efficient for large-scale sensitivity analyses.

The Bootstrap method employs non-parametric resampling of the observed data (with replacement) to capture sampling variability. For each of 1,000 bootstrap iterations, the Poisson model was refitted to the resampled dataset, and forecasts were generated to 2040. The 2.5th and 97.5th percentiles of the bootstrapped forecasts define the empirical 95% interval, which naturally incorporates model re-estimation uncertainty.

The Monte Carlo simulation method generates parameter uncertainty by sampling model coefficients from a multivariate normal distribution defined by the estimated coefficients and their variance–covariance matrix. Each of 10,000 simulations produces a possible forecast trajectory, from which the central 95% range is extracted. Monte Carlo and Bootstrap yielded nearly identical forecast intervals, confirming the model’s stability and minimal sensitivity to random perturbations.

The Bayesian posterior predictive approach incorporates both parameter and predictive uncertainty by estimating a Bayesian Poisson regression with weakly informative priors. Posterior samples were drawn using Markov Chain Monte Carlo (MCMC), and the posterior predictive distribution of future CIR values was generated to produce 95% credible intervals. These intervals were slightly wider, reflecting epistemic uncertainty in parameter estimation, although the posterior mean closely followed the frequentist forecasts.

Across all methods, the forecast trajectories were consistent and overlapping, indicating robust model specification and stable temporal patterns. The Delta-based intervals were ultimately retained in the main analysis for interpretability and computational efficiency, while this figure provides a full comparative uncertainty assessment.

**Supplemental Table 1: Proportion of SMuRF-less AMI out of total AMI from 2025 to 2040.**

|  | **2025** | **2030** | **2035** | **2040** |
| --- | --- | --- | --- | --- |
| **Singapore** |  |  |  |  |
| Overall cases | 5.8 (5.2-6.4) | 6.6 (5.5-7.6) | 7.4 (5.8-9.1) | 8.5 (6.2-10.8) |
| Overall mortality | 6.3 (5.7-6.9) | 7.8 (6.8-8.9) | 10.0 (8.4-11.6) | 13.3 (10.9-15.6) |
| **UK** |  |  |  |  |
| Overall cases | 13.8 (13.0-14.6) | 14.7 (13.6-15.8) | 15.7 (14.2-17.2) | 16.9 (15.1-18.7) |
| Overall mortality | 11.3 (10.5-12.1) | 10.7 (9.5-11.8) | 10.1 (8.6-11.6) | 9.5 (7.7-11.4) |

Abbreviations: SMuRF-less – absence of standard modifiable risk factors, AMI – acute myocardial infarction, UK – United Kingdom

* Values are presented as percentage (95% confidence interval)

* Proportion calculated using formula:

$Proportion \left( \% \right)= \frac{Crude SMuRF-less AMI cases or mortality}{Crude total AMI cases or mortality}\times100\%$.

**Supplemental Table 2: Projected SMuRF-less AMI prevalence from 2025 to 2040, stratified by sex and age category.**

|  | **2025** | **2030** | **2035** | **2040** | **Compound annual growth rate (%)** |
| --- | --- | --- | --- | --- | --- |
| **Singapore SMuRF-less** | | | | |  |
| Overall | 5.8 (5.2-6.4; N=1073) | 6.6 (5.5-7.6; N=1627) | 7.4 (5.8-9.1; N=2475) | 8.5 (6.2-10.8; N=3771) | 2.6 (2.0-3.2) |
| Male | 4.9 (4.3-5.5; N=607) | 5.7 (4.7-6.8; N=960) | 6.7 (5.0-8.4; N=1518) | 7.8 (5.4-10.3; N=2400) | 3.2 (2.6-3.8) |
| Female | 7.6 (6.6-8.6; N=465) | 8.3 (6.7-9.9; N=667) | 9 (6.7-11.4; N=956) | 9.8 (6.6-13.1; N=1371) | 1.8 (1.2-2.4) |
| Young  adults | 19.7 (12.6-26.8; N=39) | 29.1 (12.7-45.6; N=66) | 43 (9.9-76.2; N=110) | 63.6 (1.5-125.7; N=185) | 8.1 (7.5-8.7) |
| Middle-aged adults | 7.7 (6.3-9.2; N=449) | 9.8 (7.1-12.5; N=693) | 12.5 (7.9-17.1; N=1071) | 15.9 (8.5-23.2; N=1655) | 4.9 (4.3-5.5) |
| Older adults | 4.5 (3.9-5.2; N=581) | 4.7 (3.8-5.6; N=857) | 4.8 (3.6-6.1; N=1262) | 5 (3.4-6.6; N=1861) | 0.6 (0.0-1.2) |
| STEMI | 6.6 (5.8-7.3; N=202) | 7.3 (5.9-8.5; N=252) | 8 (6-9.8; N=315) | 8.8 (6-11.4; N=392) | 2.0 (1.4-2.6) |
| NSTEMI | 5.5 (4.7-6.3; N=870) | 6.2 (4.9-7.6; N=1438) | 7.1 (5-9.2; N=2377) | 8 (5.1-11.1; N=3929) | 2.6 (2.0-3.2) |
| Overweight/obesity | 2.5 (1.8-3.3; N=470) | 3.1 (1.8-4.1; N=768) | 3.7 (1.8-5.7; N=1244) | 4.5 (1.7-7.3, N=1999) | 3.9 (3.3-4.5) |
| **UK SMuRF-less** |  |  |  |  |  |
| Overall | 13.8 (13.0-14.6; N=7427) | 14.7 (13.6-15.8; N=7809) | 15.7 (14.2-17.2; N=8272) | 16.9 (15.1-18.7; N=8820) | 1.3 (0.5-2.1) |
| Male | 14.2 (13.6-14.8; N=5378) | 15.6 (14.6-16.4; N=5956) | 17.1 (15.6-18.3; N=6596) | 18.7 (16.7-20.4; N=7304) | 1.8 (1.0-2.6) |
| Female | 12.9 (12.2-13.6; N=2049) | 12.4 (11.5-13.4; N=1853) | 12 (10.9-13.2; N=1676) | 11.6 (10.2-12.9; N=1515) | -0.7 (-1.5-0.1) |
| Young adults | 17.7 (14.4-20.9; N=117) | 18.9 (13.7-24.1; N=125) | 20.3 (12.8-27.7; N=133) | 21.8 (11.6-31.8; N=142) | 1.4 (0.6-2.2) |
| Middle-aged adults | 15 (13.9-16.1; N=3139) | 16.9 (14.9-18.6; N=3691) | 18.9 (16-21.4; N=4339) | 21.2 (17.2-24.7; N=5102) | 2.3 (1.5-3.1) |
| Older adults | 12.8 (12.5-13.1; N=4003) | 12.8 (12.3-13.2; N=3833) | 12.8 (12.2-13.3; N=3671) | 12.8 (12-13.5; N=3515) | 0.0 (-0.8-0.8) |
| STEMI | 17.1 (15.4-18.7; N=3024) | 18.6 (16-21.2; N=3177) | 20.3 (16.6-23.9; N=3338) | 22.1 (17.2-27.0; N=3508) | 1.7 (0.9-2.5) |
| NSTEMI | 11.8 (11.4-12.3; N=4255) | 12 (11.3-12.6; N=4304) | 12.1 (11.2-12.9; N=4353) | 12.2 (11.2-13.3; N=4403) | 0.2 (-0.6-1.0) |
| Overweight/obesity | 12.8 (11.8-13.7; N=6851) | 14.2 (12.8-15.5; N=7517) | 15.8 (14.0-17.6; N=8293) | 17.6 (15.4-19.8; N=9196) | 2.2 (1.4-3.0) |

Abbreviations: AMI – acute myocardial infarction, SMuRF-less – absence of standard modifiable risk factors, UK – United Kingdom, STEMI – ST-elevation myocardial infarction, NSTEMI – non-ST elevation myocardial infarction

* Values are presented as prevalence rate (95% confidence interval; N=number of cases). Values for compound annual growth rate are presented as percentage (95% confidence interval).

* SMuRF-less AMI prevalence (%) calculated using formula:

$$SMuRF-less AMI prevalence= \frac{Crude SMuRF-less AMI cases}{Crude total AMI cases} \times100\%$$

* Compound annual growth rate (CAGR) calculated using formula:

$$CAGR=\left( \left( \frac{EV}{BV} \right)^{\frac{1}{n}}-1 \right)\times100$$

where *EV*=ending value, *BV*=beginning value and *n*=number of years​.

* Forecast estimates for specific demographic subgroups with relatively low numbers of AMI cases, particularly younger adults aged 15–39 years, should be interpreted with caution. The relatively low numbers of AMI cases may introduce greater statistical uncertainty and instability in model projections. Although Poisson regression models were used with robust variance estimation, the limited event counts can inflate compound annual growth rates and widen confidence intervals. As such, 95% confidence intervals for forecasted proportions have been included.

**Supplemental Figure 2: Projected total AMI prevalence in Singapore and UK from 2025 to 2040. Bar charts depict crude AMI cases and line graphs depict AMI prevalence.**
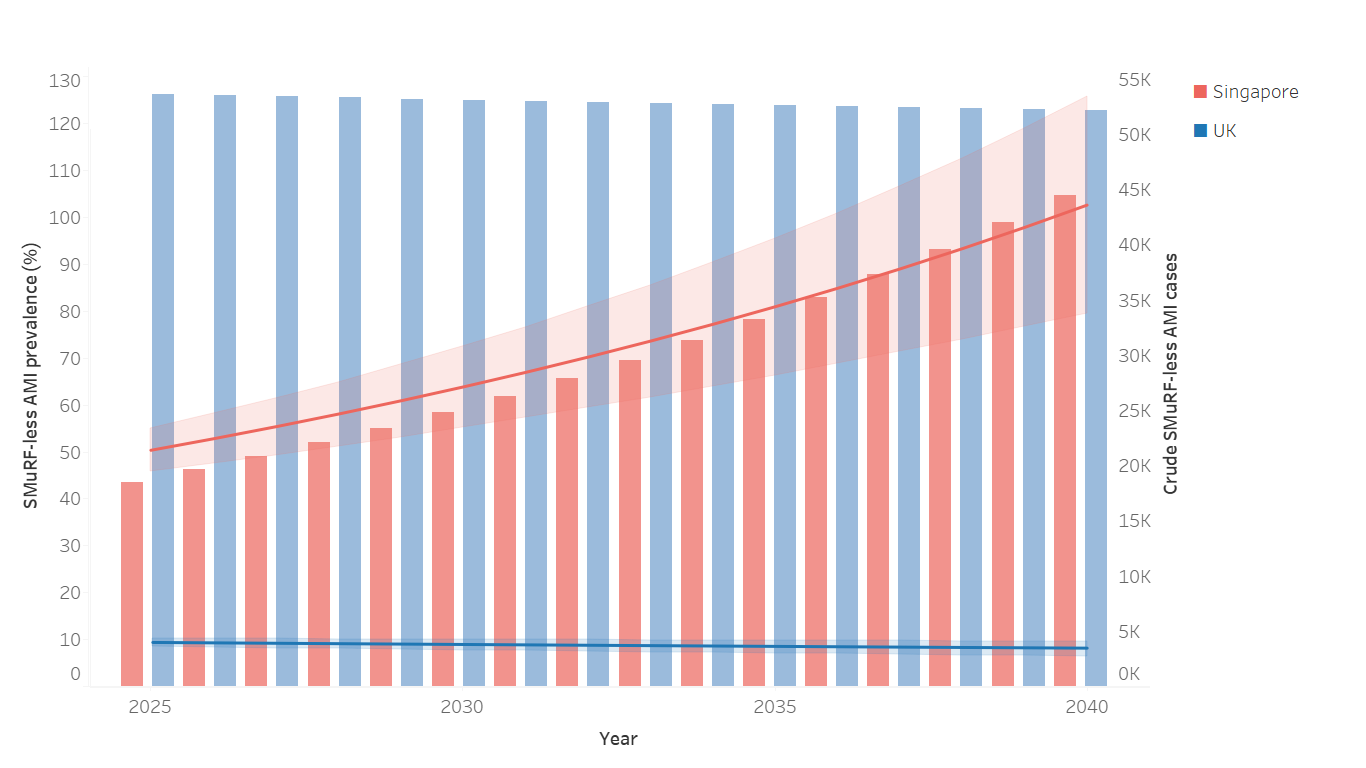


Abbreviations: AMI – acute myocardial infarction, UK – United Kingdom

**Supplemental Figure 3: Proportion of projected SMuRF-less AMI prevalence and mortality out of total AMI in Singapore and UK from 2025 to 2040.**

A) Prevalence

**
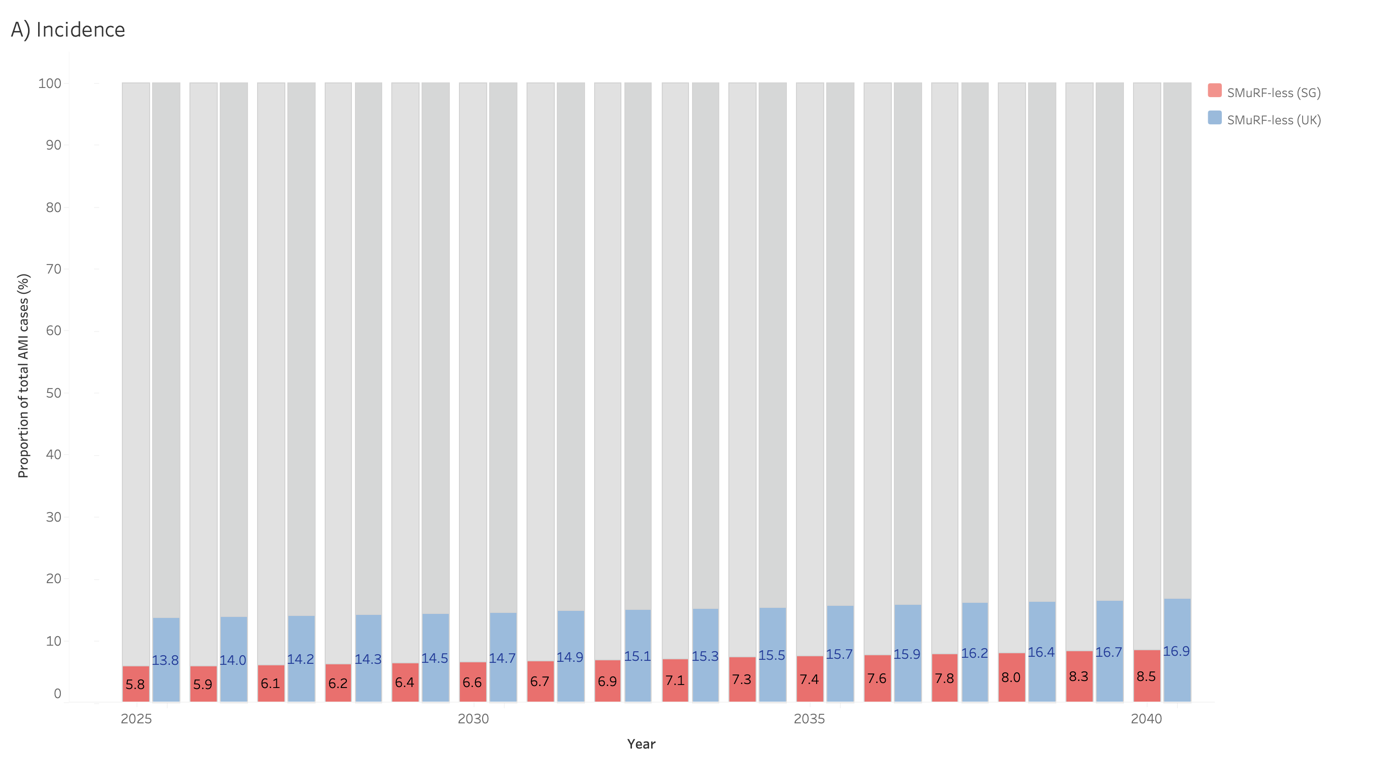
**

B) Mortality
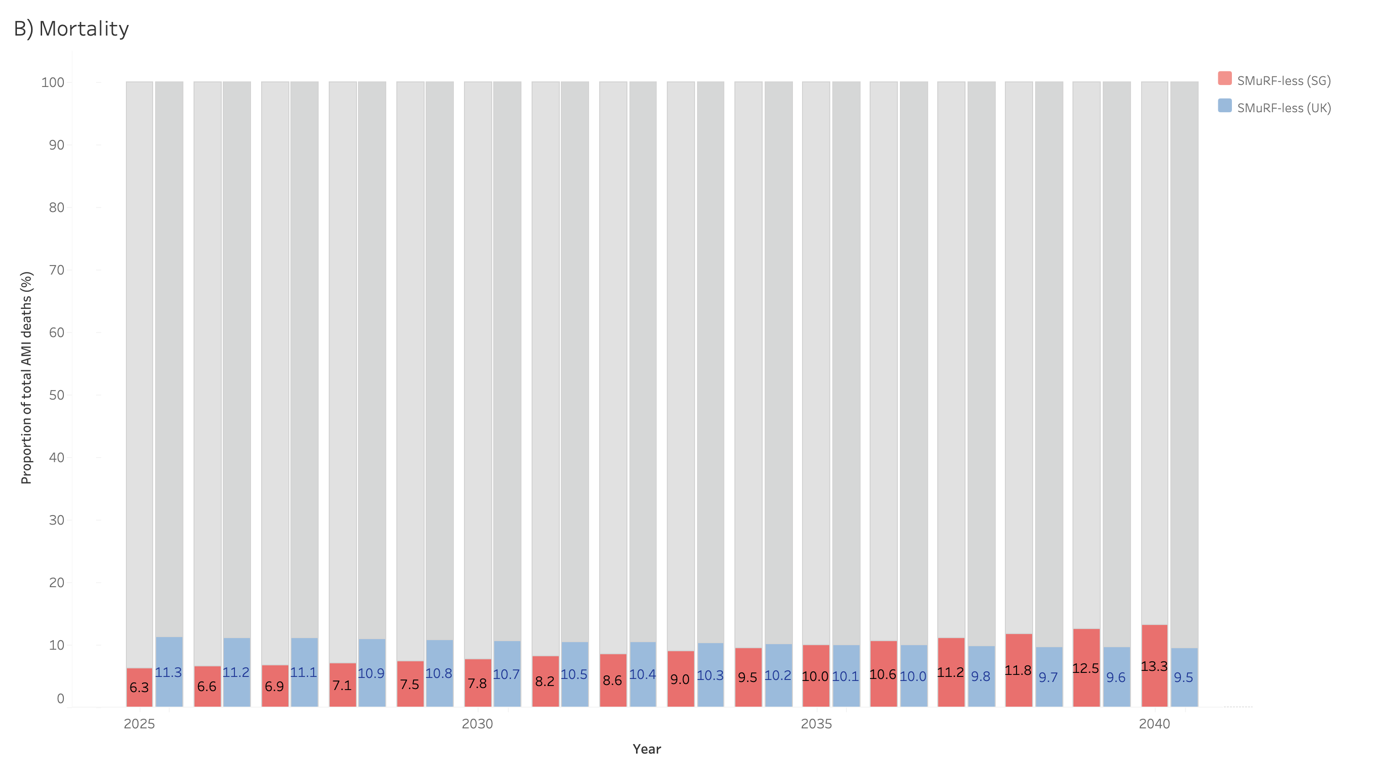


Abbreviations: SMuRF-less – absence of standard modifiable risk factors, AMI – acute myocardial infarction, SG – Singapore, UK – United Kingdom

**Supplemental Table 3: Projected SMuRF-less AMI case fatality rate from 2025 to 2040, stratified by sex and age category.**

|  | **2025** | **2030** | **2035** | **2040** | **Compound annual growth rate (%)** |
| --- | --- | --- | --- | --- | --- |
| **Singapore SMuRF-less** | | | |  |  |
| Overall | 5.1 (3.7-6.4; N=54) | 4.2 (3.2-5.2; N=69) | 3.7 (2.9-4.4; N=91) | 3.3 (2.7-3.9; N=123) | -2.9 (-3.5--2.3) |
| Male | 6.5 (1.8-5.1; N=40) | 5.9 (1.2-6.2; N=56) | 5.3 (0.7-7.3; N=81) | 4.8 (0.1-8.7; N=115) | -2.0 (-2.6--1.4) |
| Female | 3.1 (0.8-3.7; N=15) | 1.8 (0.4-2.5; N=12) | 1.1 (0.2-1.7; N=10) | 0.6 (0.1-1.2; N=9) | -10.2 (-10.8--9.6) |
| Middle-aged adults | 3.8 (3.4-7; N=17) | 3.6 (5-11.3; N=25) | 3.4 (7.2-17.4; N=36) | 3.2 (10.4-26; N=52) | -1.2 (-1.8--0.6) |
| Older adults | 5.3 (3.5-21.7; N=31) | 3.6 (2.6-21.4; N=31) | 2.4 (1.9-21.1; N=30) | 1.6 (1.4-20.7; N=30) | -7.8 (-8.4--7.2) |
| STEMI | 10.9 (11.8-15.4; N=22) | 10.4 (14.5-18.4; N=26) | 10 (17.3-22; N=31) | 9.6 (20.4-26.4; N=38) | -0.8 (-1.4--0.2) |
| NSTEMI | 0.9 (0.7-5.5; N=8) | 0.4 (0.4-4.4; N=6) | 0.2 (0.2-3.4; N=5) | 0.1 (0.1-2.6; N=4) | -14.3 (-14.9--13.7) |
| Overweight/obesity | 3.2 (2.5-3.9; N=15) | 2.4 (1.2-3.7; N=19) | 1.9 (0.0-3.8; N=23) | 1.5 (0.0-4.3; N=30) | -5.0 (-5.6--4.4) |
| **UK SMuRF-less** |  |  |  |  |  |
| Overall | 5.5 (4.8-6.2; N=406) | 4.9 (4.4-5.4; N=384) | 4.4 (4-4.9; N=366) | 4 (3.6-4.4; N=351) | -2.1 (-2.9--1.3) |
| Male | 4.5 (3.7-5.3; N=242) | 4.1 (1.4-11.7; N=244) | 3.7 (0.3-13.2; N=246) | 3.4 (0.1-14.7; N=248) | -1.9 (-2.7--1.1) |
| Female | 8.0 (7.2-8.8; N=164) | 7.6 (6.8-12.1; N=140) | 7.1 (5.4-11.8; N=120) | 6.8 (4.3-11.5; N=102) | -1.1 (-1.9--0.3) |
| Middle-aged adults | 3.9 (3.2-4.7; N=124) | 4.8 (3.9-12.1; N=179) | 5.9 (3.7-18.1; N=258) | 7.3 (2.9-26.9; N=372) | 4.2 (3.4-5.0) |
| Older adults | 10.6 (10.0-11.2; N=425) | 10.4 (10.9-15.8; N=399) | 10.2 (10-16.2; N=376) | 10 (9.1-16.7; N=353) | -0.4 (-1.2-0.4) |
| STEMI | 11.1 (10.3-11.9; N=336) | 12.3 (16.4-29.2; N=390) | 13.6 (17.1-37.7; N=453) | 15 (17.4-48.5; N=526) | 2.0 (1.2-2.8) |
| NSTEMI | 4 (3.2-4.8; N=168) | 3.3 (2.4-5.8; N=144) | 2.8 (1.6-5.4; N=123) | 2.4 (1-5.1; N=105) | -3.3 (-4.1--2.5) |
| Overweight/obesity | 5.6 (4.7-6.6; N=386) | 5.6 (4.3-7.0; N=424) | 5.7 (3.9-7.5; N=476) | 5.9 (3.7-8.1; N=5421) | 0.3 (-0.5-1.1) |

Abbreviations: AMI – acute myocardial infarction, SMuRF-less – absence of standard modifiable risk factors, UK – United Kingdom, STEMI – ST-elevation myocardial infarction, NSTEMI – non-ST elevation myocardial infarction

* Values are presented as mortality rate (95% confidence interval; N=number of deaths). Values for compound annual growth rate are presented as percentage (95% confidence interval).

* Case fatality rate (%) calculated using formula:

$Case fatality rate= \frac{Crude SMuRF-less mortality}{Crude SMuRF-less cases} \times100\%$

* Compound annual growth rate (CAGR) calculated using formula:

$CAGR=\left( \left( \frac{EV}{BV} \right)^{\frac{1}{n}}-1 \right)\times100$
where *EV*=ending value, *BV*=beginning value and *n*=number of years​.

* Case fatality trends in the age 15-39 years category was not forecasted due to the low number of mortality cases.

**Supplemental Figure 4. Projected SMuRF-less AMI case fatality rate from 2025 to 2040, stratified by (A) Male and, (B) Female. Bar charts depict crude AMI mortality and line graphs depict case fatality rate of AMI.**
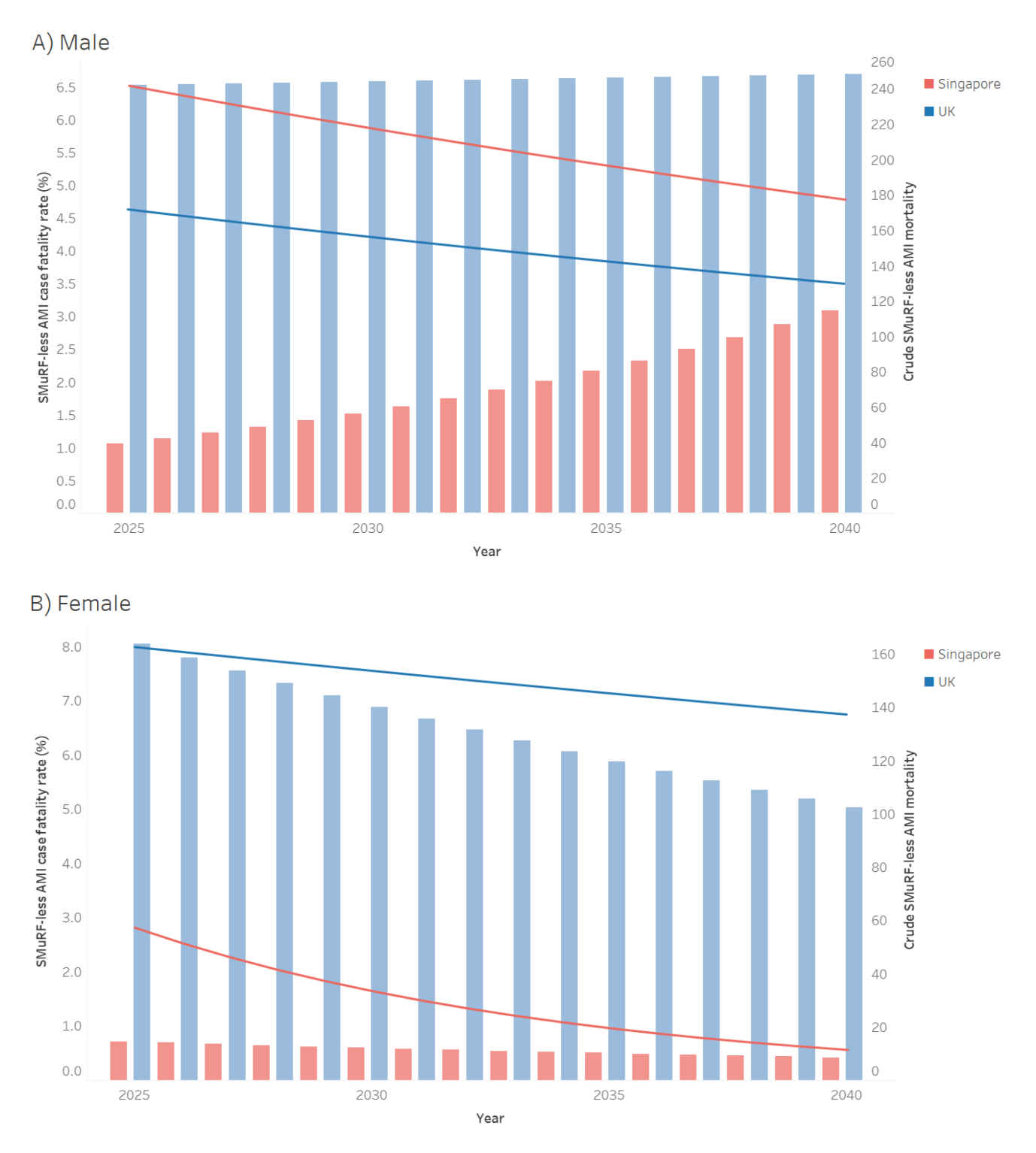


Abbreviations: SMuRF-less – absence of standard modifiable risk factors, AMI – acute myocardial infarction, UK – United Kingdom

**Supplemental Figure 5. Proportion of projected SMuRF-less AMI cases from 2025 to 2040, stratified by age category in (A) Singapore and, (B) UK.**

**
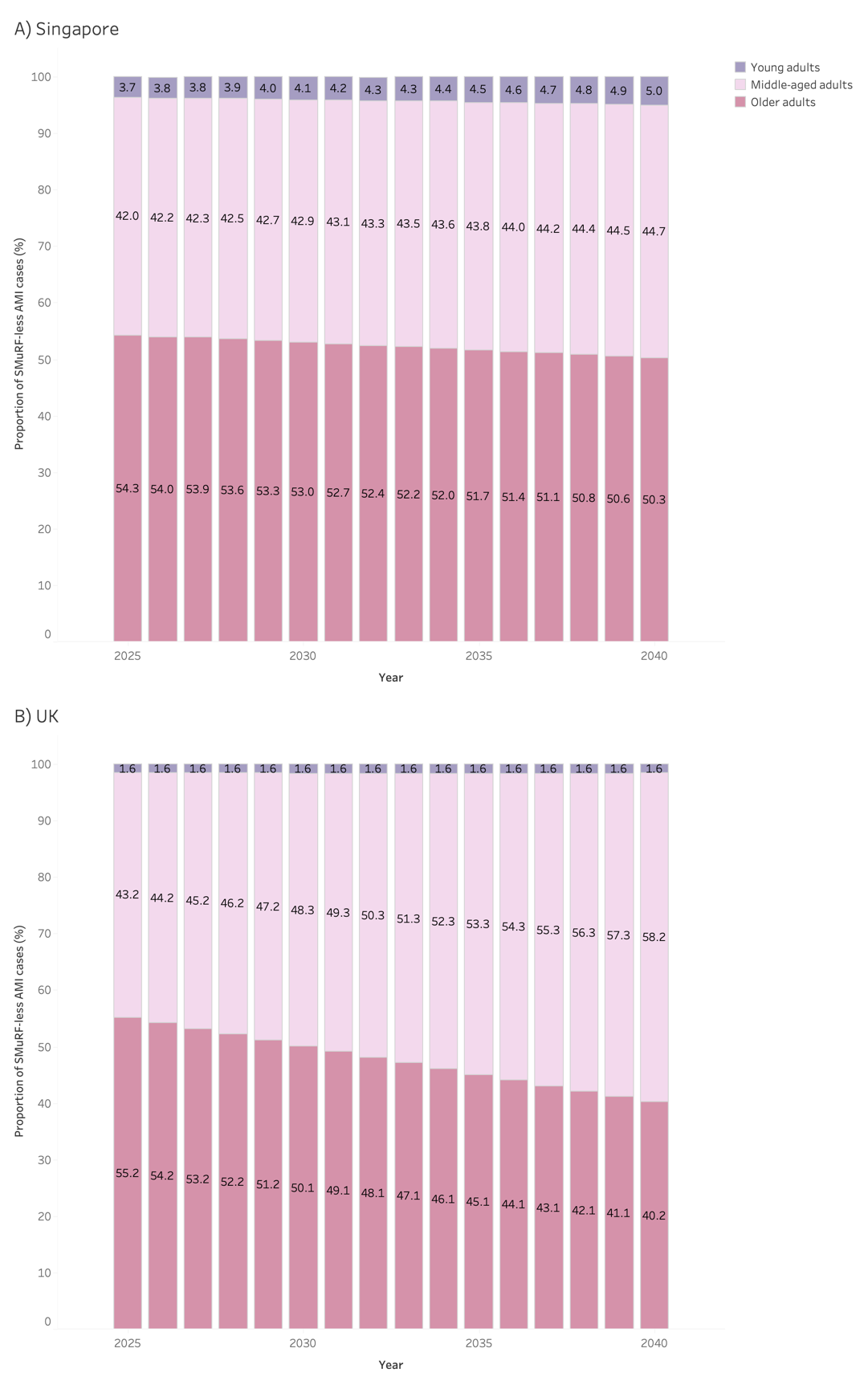
**

Abbreviations: SMuRF-less – absence of standard modifiable risk factors, AMI – acute myocardial infarction, UK – United Kingdom

**Supplemental Figure 6. Projected SMuRF-less AMI case fatality rate from 2025 to 2040, stratified by (A) Middle-aged adults and, (B) Older adults. Bar charts depict crude AMI mortality and line graphs depict case fatality rate of AMI.
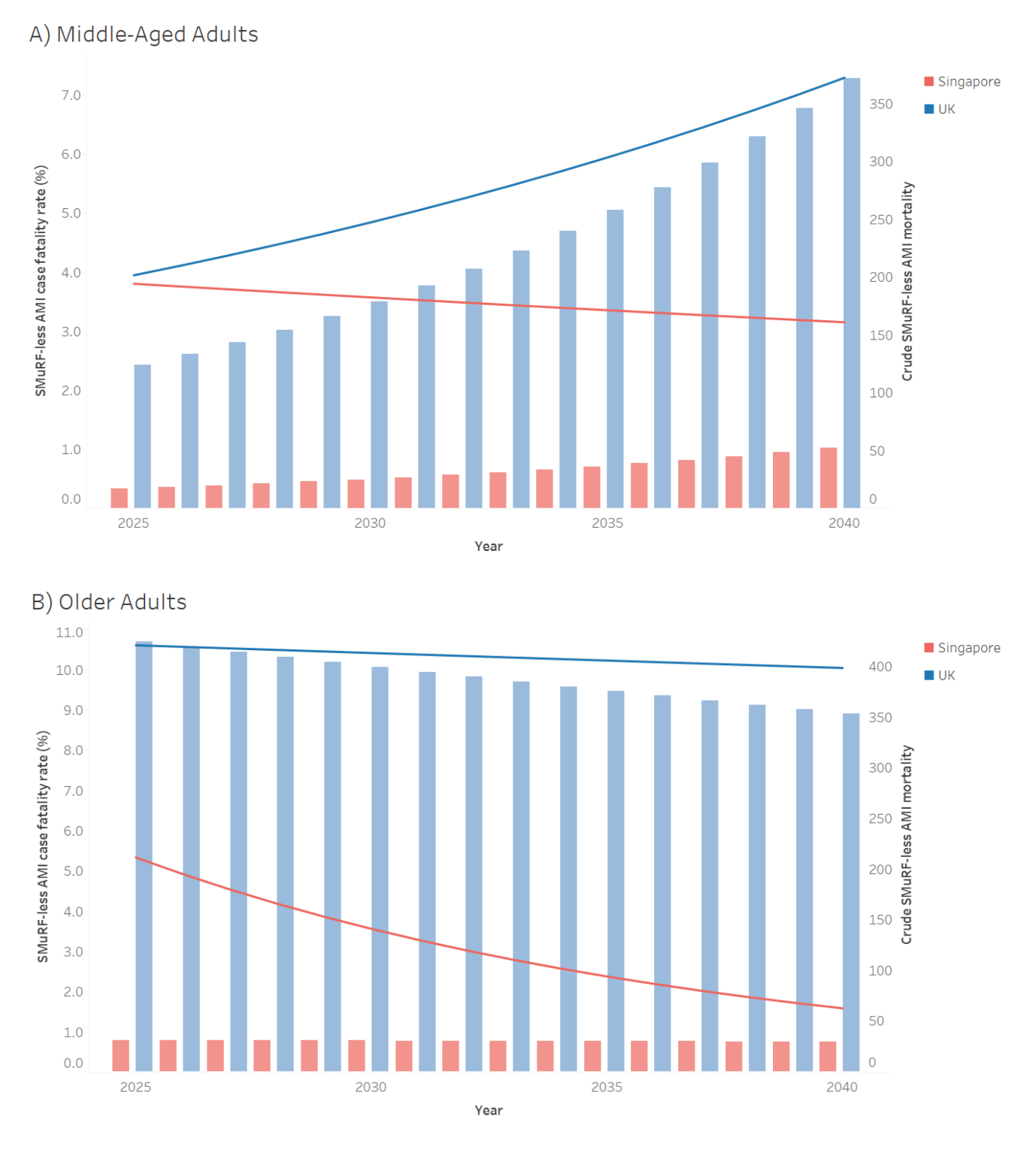
**

Abbreviations: SMuRF-less – absence of standard modifiable risk factors, AMI – acute myocardial infarction, UK – United Kingdom

**Supplemental Figure 7. Projected SMuRF-less AMI case fatality rate from 2025 to 2040, stratified by (A) STEMI and, (B) NSTEMI. Bar charts depict crude AMI mortality and line graphs depict case fatality rate of AMI.
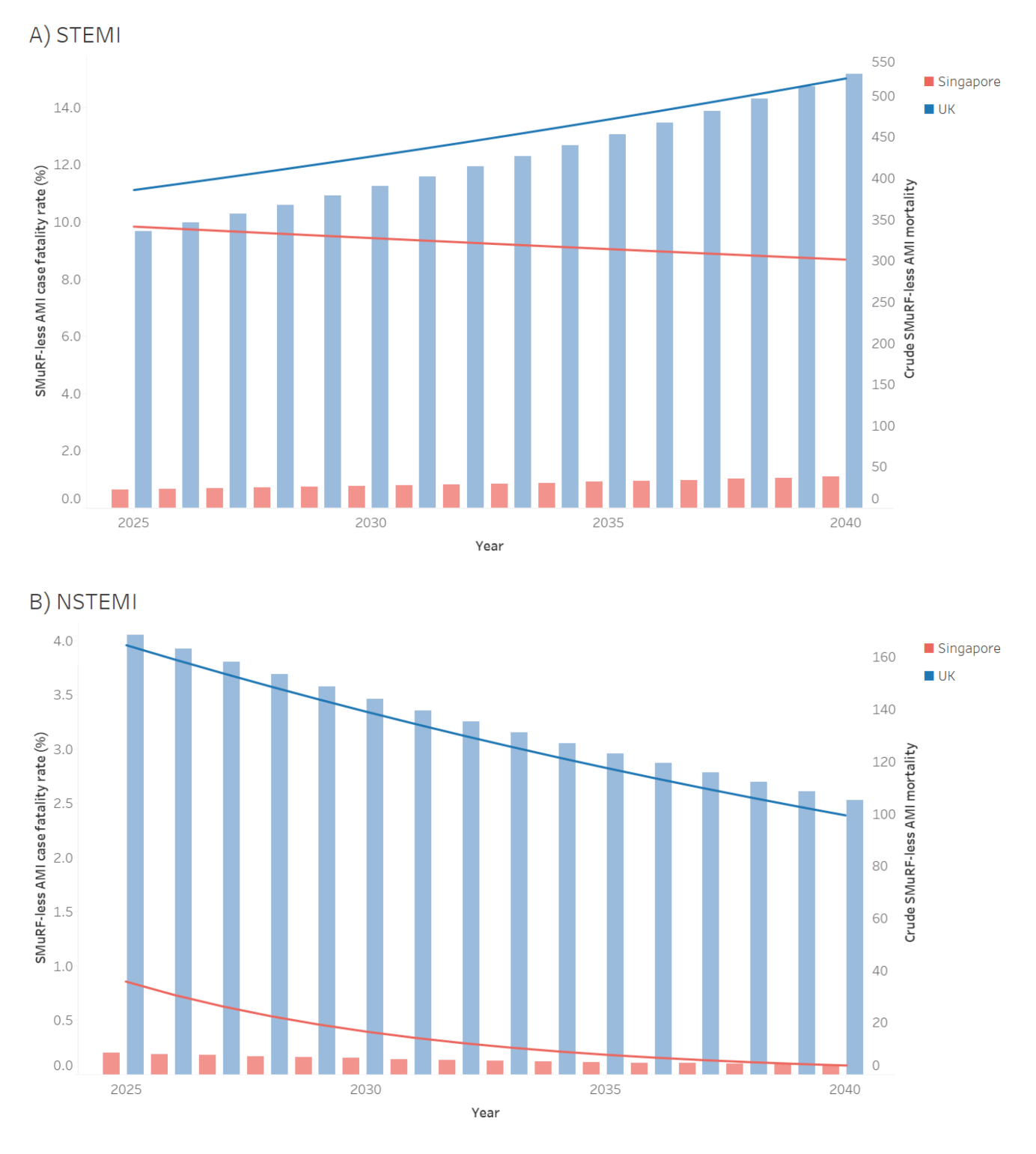
**

Abbreviations: SMuRF-less – absence of standard modifiable risk factors, AMI – acute myocardial infarction, UK – United Kingdom, STEMI – ST-elevation myocardial infarction, NSTEMI – non-ST elevation myocardial infarction

**Supplemental Figure 8: (A) Projected SMuRF-less AMI prevalence and, (B) Projected SMuRF-less AMI case fatality rate in overweight/obesity population from 2025 to 2040. Bar charts depict crude SMuRF-less overweight/obesity AMI cases or mortality and line graphs depict SMuRF-less overweight/obesity AMI prevalence or case fatality rate.
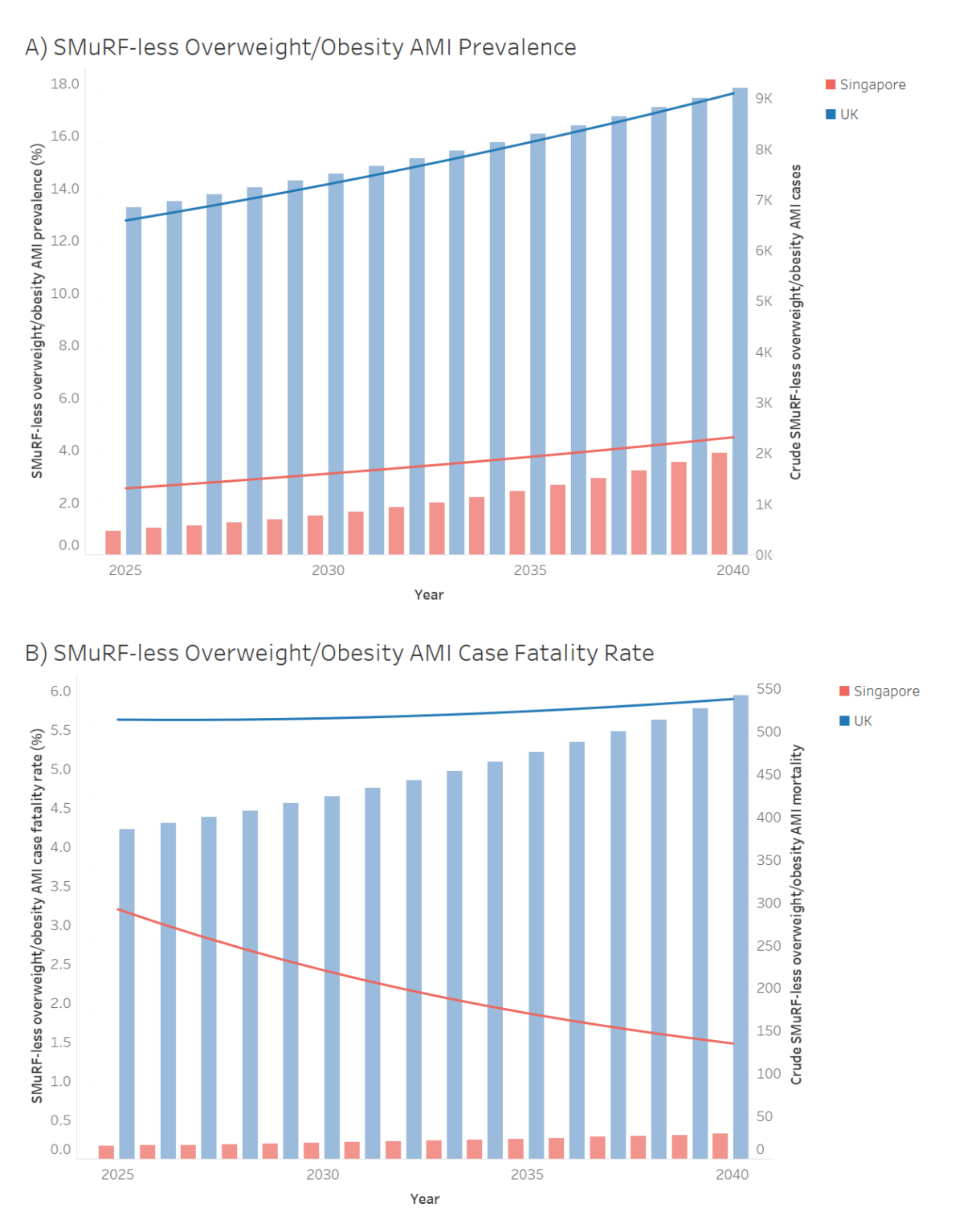
**

Abbreviations: AMI – acute myocardial infarction, SMuRF-less – absence of standard modifiable risk factors

**Supplemental Figure 9: (A) Proportion of projected SMuRF-less AMI cases and, (B) Proportion of projected SMuRF-less AMI mortality in overweight/obesity population in Singapore and UK from 2025 to 2040, stratified by sex.**
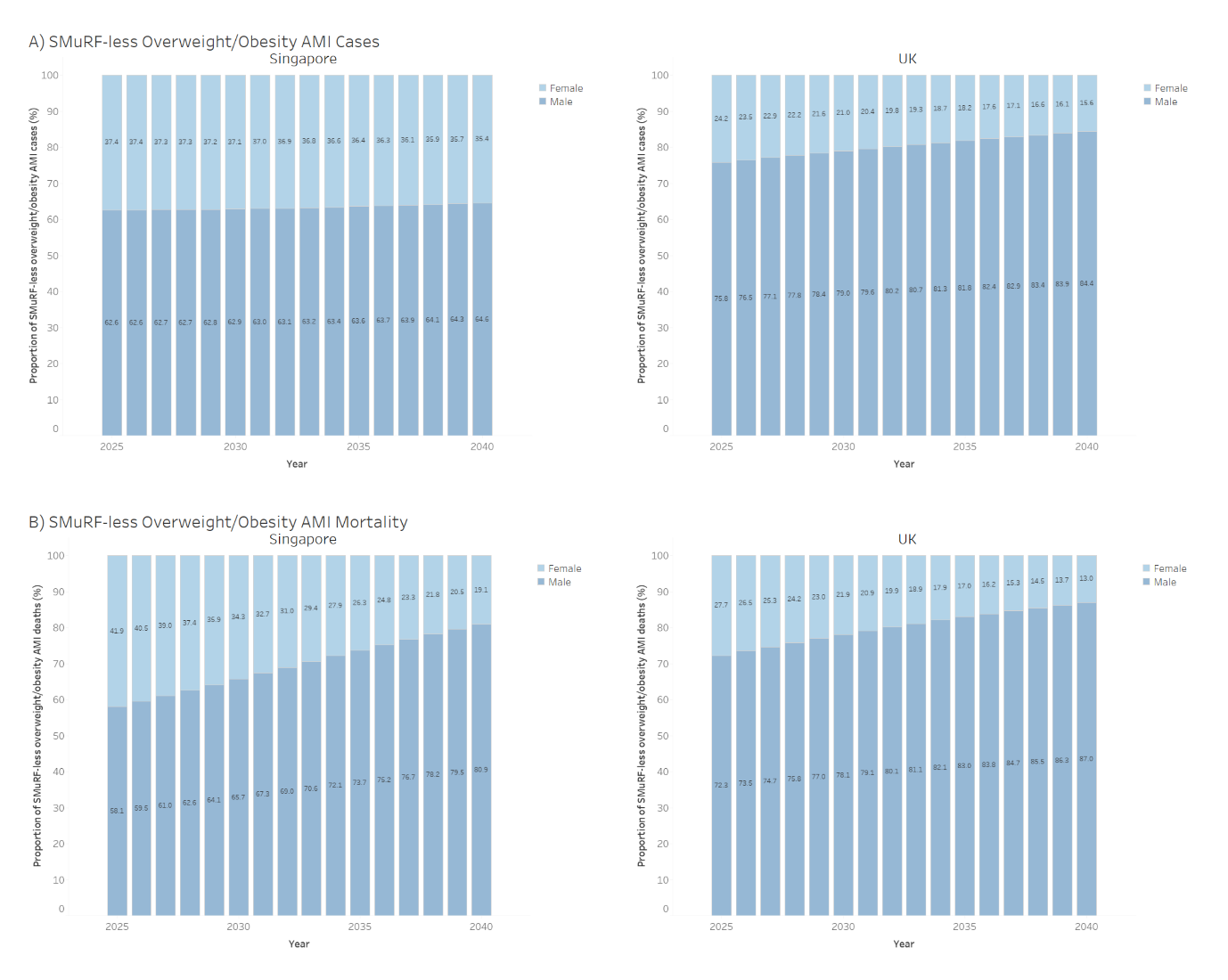
Abbreviations: SMuRF-less – absence of standard modifiable risk factors, AMI – acute myocardial infarction, UK – United Kingdom

**Supplemental Figure 10: Proportion of projected SMuRF-less AMI cases in overweight/obesity population from 2025 to 2040, stratified by age category in (A) Singapore and, (B) UK.**
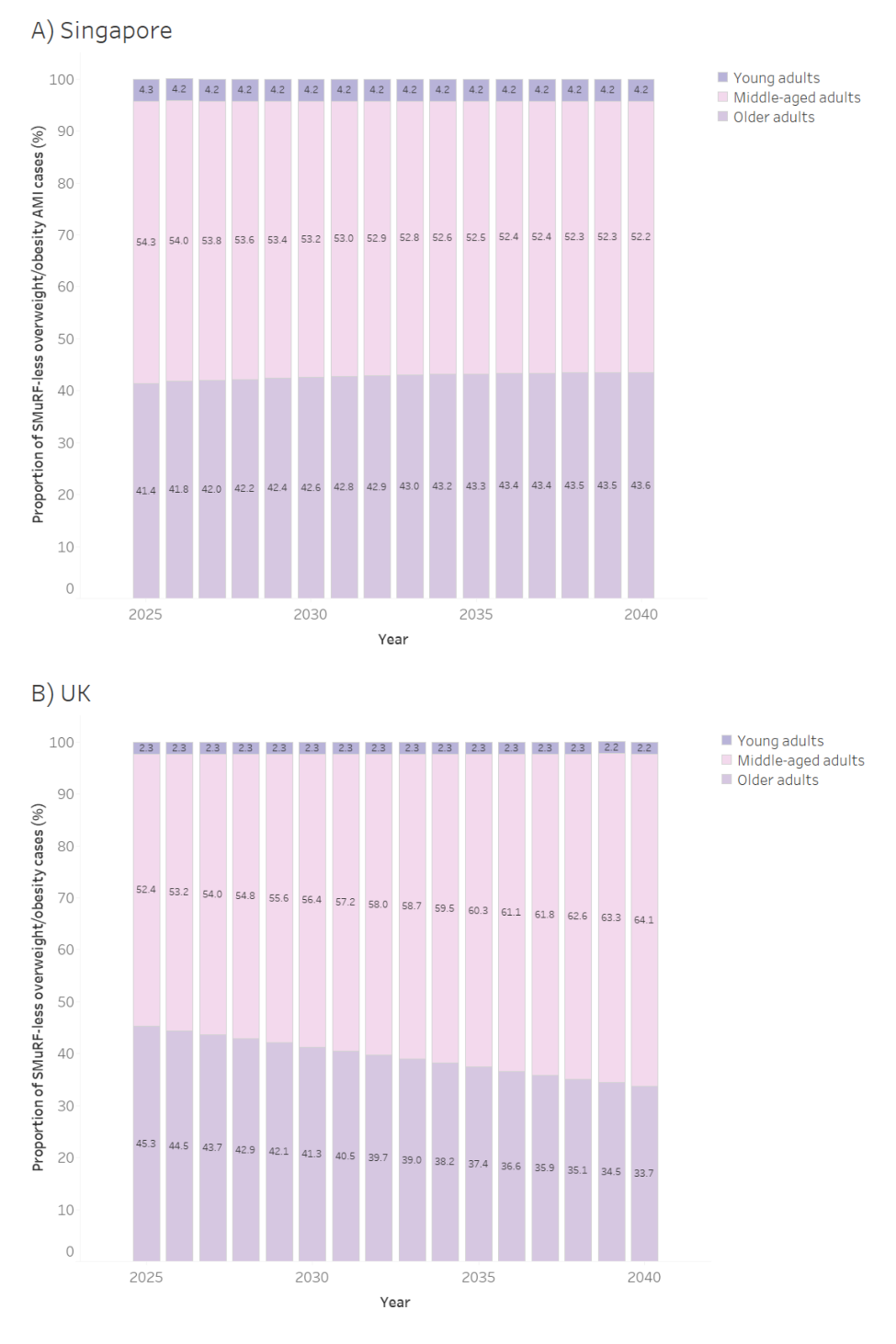


Abbreviations: SMuRF-less – absence of standard modifiable risk factors, AMI – acute myocardial infarction, UK – United Kingdom

**REFERENCES**

1. Arnett DK, Blumenthal RS, Albert MA, Buroker AB, Goldberger ZD, Hahn EJ, et al. 2019 ACC/AHA Guideline on the Primary Prevention of Cardiovascular Disease: A Report of the American College of Cardiology/American Heart Association Task Force on Clinical Practice Guidelines. Circulation. 2019;140(11):e596-e646.

2. Goh SY, Ang SB, Bee YM, Chen YT, Gardner DS, Ho ET, et al. Ministry of Health Clinical Practice Guidelines: Diabetes Mellitus. Singapore Med J. 2014;55(6):334-47.

3. Tay JC, Sule AA, Chew EK, Tey JS, Lau T, Lee S, et al. Ministry of Health Clinical Practice Guidelines: Hypertension. Singapore Med J. 2018;59(1):17-27.

4. Whelton PK, Carey RM, Mancia G, Kreutz R, Bundy JD, Williams B. Harmonization of the American College of Cardiology/American Heart Association and European Society of Cardiology/European Society of Hypertension Blood Pressure/Hypertension Guidelines: Comparisons, Reflections, and Recommendations. Circulation. 2022;146(11):868-77.

5. Tai ES, Chia BL, Bastian AC, Chua T, Ho SC, Koh TS, et al. Ministry of Health Clinical Practice Guidelines: Lipids. Singapore Med J. 2017;58(3):155-66.

6. Grundy SM, Stone NJ, Bailey AL, Beam C, Birtcher KK, Blumenthal RS, et al. 2018 AHA/ACC/AACVPR/AAPA/ABC/ACPM/ADA/AGS/APhA/ASPC/NLA/PCNA Guideline on the Management of Blood Cholesterol: Executive Summary: A Report of the American College of Cardiology/American Heart Association Task Force on Clinical Practice Guidelines. J Am Coll Cardiol. 2019;73(24):3168-209.

7. Moledina SM, Rashid M, Nolan J, Nakao K, Sun LY, Velagapudi P, et al. Addressing disparities of care in non-ST-segment elevation myocardial infarction patients without standard modifiable risk factors: insights from a nationwide cohort study. European Journal of Preventive Cardiology. 2021;29(7):1084-92.

8. Wilkinson C, Weston C, Timmis A, Quinn T, Keys A, Gale CP. The Myocardial Ischaemia National Audit Project (MINAP). Eur Heart J Qual Care Clin Outcomes. 2020;6(1):19-22.

9. Azur MJ, Stuart EA, Frangakis C, Leaf PJ. Multiple imputation by chained equations: what is it and how does it work? Int J Methods Psychiatr Res. 2011;20(1):40-9.
